# F-box/LRR-repeat protein 12 reorchestrated microglia to inhibit scarring and achieve adult spinal cord injury repair

**DOI:** 10.1038/s41392-025-02354-0

**Published:** 2025-08-20

**Authors:** Xu Xu, Feng Gao, Qixin Chen, Bairu Chen, Wenyu Liang, Runzhi Huang, Yuchen Liu, Zhibo Liu, Yanjing Zhu, Gufa Lin, Bei Ma, Letao Yang, Shaorong Gao, Rongrong Zhu, Liming Cheng

**Affiliations:** 1 Key Laboratory of Spine and Spinal Cord Injury Repair and Regeneration of Ministry of Education, Department of Orthopedics, School of Medicine, Tongji Hospital affiliated with Tongji University, School of Life Science and Technology, Tongji University, Shanghai, China; 2 Frontier Science Center for Stem Cell Research, Tongji University, Shanghai, China; 3 Clinical Center for Brain and Spinal Cord Research, Tongji University, Shanghai, China

**Keywords:** Regeneration and repair in the nervous system, Molecular neuroscience

## Abstract

Scarring is an insurmountable obstacle for axonal regeneration in recovery from spinal cord injury (SCI). It impedes the repair effects of therapeutic targets in cortical neurons, such as PTEN^−/−^ and hyper-IL-6, which cannot break through dense scar barriers to reconstruct neural circuits. However, methods for eliminating this process remain elusive. Here, we conducted a multiomics analysis of SCI and identified FBXL12 as an effective target for inhibiting scarring, further promoting spontaneous crossing of axons at the epicenter. We identified N6-Methyladenosine (m6A) modification as the predominant mRNA modification in SCI, with Fbxl12 being a major modification target. Furthermore, m6A modification specifically promoted FBXL12 synthesis in activated microglia. The overexpression of FBXL12 in microglia contributed to its homogeneous distribution and maintained a “scar-less healing” phenotype. Remarkably, FBXL12 therapy effectively reduced extracellular matrix deposition and decreased the scar area by ~70%. Importantly, axons grew through the epicenter and reached a length of more than 2.4 mm 56 days post-SCI, significantly improving motor function and reconstructing the neural circuit. Mechanistically, FBXL12 promoted cytoskeletal reorganization and migration in microglia by catalyzing the K63-linked ubiquitylation of Myosin heavy chain 14 (MYH14). Together, our results identify m6A-FBXL12-MYH14 axis as a novel cytoskeletal reorganization pathway in activated microglia and suggest FBXL12 as an effective target for a novel microglia-based approach to facilitate scarless functional recovery in SCI.

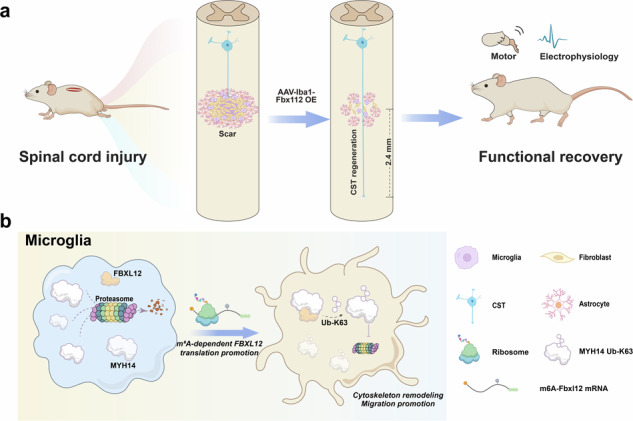

## Introduction

As a devastating injury, spinal cord injury (SCI) has an immediate impact on the sensory, motor, and autonomic functions of patients. All neuronal connections across the transection region were cut off by physically complete lesions spanning the entire width of the spinal cord.^1^ Owing to the limited regenerative ability of the central nervous system (CNS) and the lack of clinical treatments to effectively improve functional recovery from SCI, these patients experience lifetime disability.^2^ The most intractable problem that limits neural regeneration is the complex and severe pathological microenvironment that persists throughout the acute and chronic phases of the injury.^3^ The deteriorated microenvironment alters the development of cells, such as astrocytes,^4^ oligodendrocytes,^5^ and microglia,^6–8^ and triggers the formation of a scar,^9^ which prevents injured axons from regenerating across the lesion.^10^ Furthermore, the tips of the axons exhibit swollen dystrophic growth cones around the scar at the injury site of the spinal cord, which persist for decades.^11^ As long-lived resident immune cells of the CNS, microglia are activated within minutes, and our previous work has shown that microglial activation emerges significantly in two waves at 3 and 14 days postinjury.^8,12^ After injury, microglia move rapidly toward the injury site for debris clearance and inflammatory regulation, including the release of chemotactic signals that guide monocyte infiltration from the circulation.^13^ Newly proliferating microglia promote wound compaction and form a compact scar around the injury site in association with reactive astrocytes, NG2 glia, fibroblasts, and macrophages.^14^

Since microglia play a key role in microenvironment regulation and scar formation, many studies have focused on the reorganization of the microglial phenotype. Transplantation of M2-Deviated or peptidase inhibitor-treated microglia improves healing and axon regrowth.^7,15^ In addition to the above work, which concerns the immune response and immune regulation of microglia, the function of microglia in scar removal provides novel potential for injured spinal cord repair and regeneration. In their study, Li et al. have detailed the role of microglia in scar-free SCI repair in neonatal mice, identifying the molecular signatures of repair-promoting microglia. They demonstrated that transplantation of neonatal microglia significantly decreased the area of scarring.^7^ However, this effect occurred only when microglia were used from newborn mice and not from two-month-old mice. This report indicates that microglia-mediated scar repair may occur only in a specific microglial population. These studies prove that microglia are an effective target cell type; however, no current intervention strategy convincingly targets microglia within the adult spinal cord, contributing to the lack of therapies aimed at inhibiting glial scar progression in individuals with SCI. Thus, developing novel targets to create these specific microglial populations or altering the microglial state in lesion sites directly is crucial for realizing scar-free repair after SCI.

As an upstream process of cell fate and microenvironmental alterations during disease, the reversible and dynamic epigenetic modification of mRNAs adds another dimension to the complicated regulation of the primary sequence.^16–19^ N6-methyladenosine (m6A) is the most prevalent posttranscriptional mRNA modification and mediates several mRNA metabolic processes.^20,21^ The critical role of m6A epitranscriptomic regulation has been revealed in several CNS diseases, including traumatic brain injury (TBI),^22^ Alzheimer’s disease (AD),^23^ and Parkinson’s disease (PD).^24^ Weng et al. reported that axonal injury could increase m6A levels and signaling to promote protein translation of regeneration-associated genes, suggesting that m6A signaling is critical for robust axon regeneration in the adult CNS.^25^ However, despite these recent advances, the mode of mRNA epigenetic modifications and the underlying mechanisms regulating cell responses during SCI progression are still not fully understood.

Therefore, to investigate the role of epitranscriptomic modifications and reveal effective targets for regulating cell responses, we performed LC‒MS-based mRNA modification analysis and transcriptional and epitranscriptomic profiling at 0–42 days postinjury. Through integrated omics analysis, we identified a key gene, FBXL12 (F-box/LRR-repeat protein 12), that regulates the pathological process of SCI. As an E3 ubiquitin ligase, FBXL12 has been reported in previous studies to play a crucial role in regulating the cell cycle in tumor cells and T cells via ubiquitination. Specifically, it targets the cyclin-dependent kinase inhibitor Cdkn1b for polyubiquitination and proteasomal degradation.^26^ We revealed an epitranscriptomic mechanism whereby SCI elevates m6A levels and signaling to promote FBXL12 protein translation in activated microglia, which is essential for motility and the secretion of chemotaxis/complement. Moreover, we demonstrated that target overexpression of Fbxl12 in microglia promotes cytoskeletal rearrangement and enhances microglial mobility while reducing the secretion of chemokines and complement proteins. We found that overexpression of FBXL12 can induce microglia to switch to a stable state that is anti-senescent and anti-inflammatory. We have also shown that targeted overexpression of Fbxl12 in microglia can achieve scarless healing, with regenerating axons crossing the injury epicenter and growing downward by more than 2.4 mm, significantly enhancing functional recovery in the injury model. These findings suggest a novel molecular target and intervention strategy for the ideal recovery of human SCI patients.

## Results

### m6A methylation of Fbxl12 is a potential key regulator during SCI progression

**Fig. 1:**
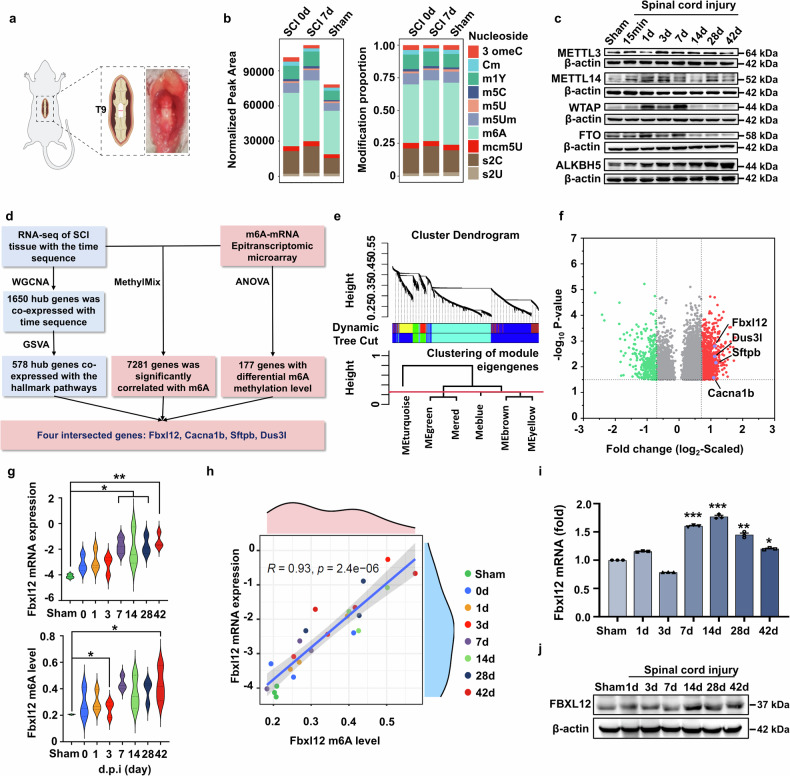
m6A methylation of Fbxl12 is a candidate master process during SCI progression. **a** Schematic illustration of the anatomical location of the C57BL/6 mouse spinal cord crush injury model. **b** Normalized peak areas and modification proportions of various mRNAs in the sham and SCI groups. The normalized peak area was calculated as the number of sites detected for this type of modification, and the modification proportion was calculated as the ratio of the detected sites for one type of modification among all detected modification sites. **c** Immunoblotting of m6A writers and erasers in spinal cord tissue at different days postinjury as indicated. **d** Schematic diagram of the analysis of integrated transcriptional and epitranscriptomic profiling. **e** Gene cluster dendrogram of the WGCNA results. **f** Volcano plot of gene expression at 7 days postinjury compared with that in the sham group. **g** mRNA expression and m6A level of Fbxl12 at different days postinjury as indicated (one-way ANOVA, *P < 0.05, **P < 0.01). **h** Correlation between Fbxl12 mRNA expression and Fbxl12 m6A levels. **i** RT‒qPCR of Fbxl12 mRNA at different days postinjury as indicated (*t* test, mean ± SEM; all groups compared with the Sham group, *P < 0.05, **P < 0.01, ***P < 0.001). **j** Immunoblotting of FBXL12 in spinal cord tissue at different days postinjury, as indicated. The results are representative of three independent experiments

Initial experiments aimed to systematically investigate the landscape of posttranscriptional modifications and potential regulators associated with SCI progression. First, we established a mouse crush injury-based SCI model (Fig. 1a) and performed RNA-seq, LC‒MS-based mRNA modification analysis and epitranscriptomic microarray of injured spinal cord tissue with a time sequence of 0–42 days. Among them, 3, 7, 14, 28, and 42 days were selected as the key time points for the progression of pathological processes in patients with SCI.^7,8,27,28^ The results revealed elevated modification levels in the SCI group relative to those in the sham control group at both 0 days postinjury and 7 days postinjury. Normalization analysis revealed that m6A was the main modification before and after SCI, and the modification sites were significantly altered before and after injury (Fig. 1b and Supplementary Fig. 1a). Furthermore, the global m6A level clearly increased in the acute stage of SCI (Supplementary Fig. 1b). Moreover, major m6A modifiers, including the methylases Mettl3, Mettl14, and Wtap and the demethylases Fto and Alkbh5, presented time-dependent alterations at the RNA and protein levels (Fig. 1c and Supplementary Fig. 1c, d). Notably, ALKBH5 expression is inversely correlated with transcriptional and translational profiles during SCI progression, a discrepancy potentially attributable to mRNA-level regulatory mechanisms or posttranslational protein modifications. Given their opposing functions in catalyzing m6A modification, these data suggest that m6A is significantly involved in the progression of SCI.

Thus, we analyzed the transcription and m6A modification data of the mice with SCI and the corresponding sham controls. In total, 41,414 transcriptional and 50,517 m6A methylation signatures were identified via RNA sequencing and m6A methylation mass spectrometry, respectively. We found that 1650 hub genes were significantly coexpressed with time, of which 578 hub genes were coexpressed with hallmark pathways originating from the MSIgDB database. Furthermore, we performed the MethlMix algorithm with our RNA-seq data and identified 7281 genes whose m6A methylation levels were significantly correlated with the mRNA expression levels and 177 genes whose m6A methylation levels were different among the different time groups. To characterize the key m6A-dependent regulators of SCI progression, we combined epitranscriptomic data with the RNA-seq data analyzed above (Fig. 1d, e and Supplementary Fig. 1e). Intriguingly, the integrated results identified four genes (*Fbxl12*, *Cacna1b*, *Sftpb*, and *Dus3I*), and Fbxl12, which encodes a substrate-recognition component of the SCF (SKP1-CUL1-F-box)-type E3 ubiquitin ligase complex, was the most significantly altered gene (Fig. 1f).^29^ We found that the mRNA expression and m6A levels of Fbxl12 were significantly elevated after SCI compared with those in the sham controls (Fig. 1g, h). Immunoblotting and quantitative polymerase chain reaction (qPCR) also confirmed a modest increase in Fbxl12 expression during SCI progression (Fig. 1I, j and Supplementary Fig. 1f). Together, these transcriptomic data suggest functional connections between m6A modifications and Fbxl12, which are implicated in the regulation of SCI progression.

### m6A promotes FBXL12 synthesis in activated microglia

**Fig. 2:**
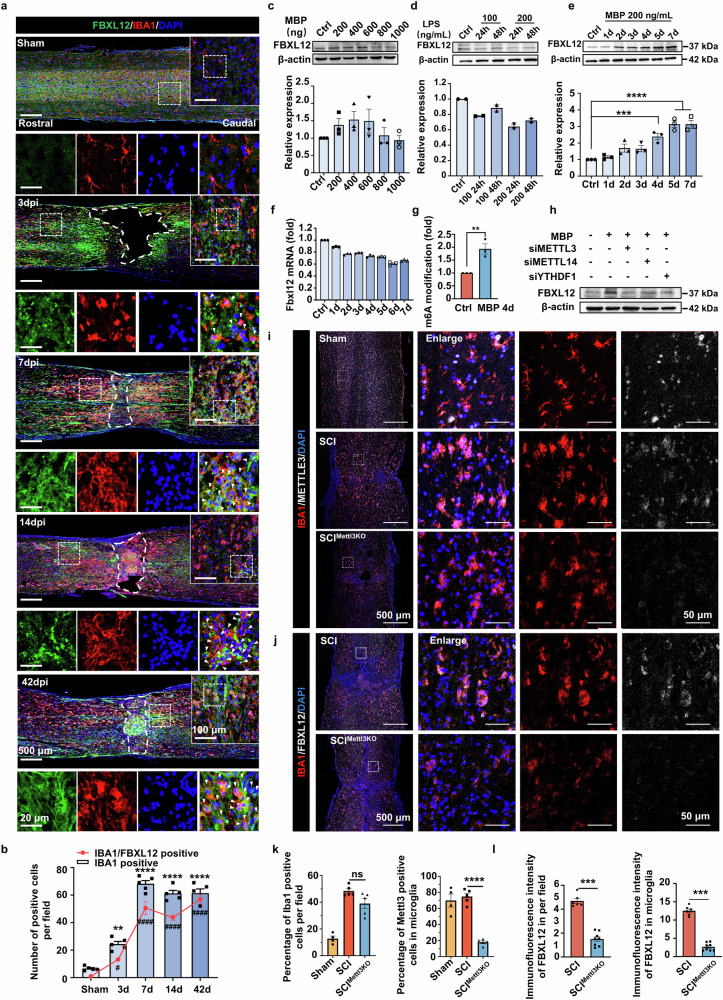
m6A promotes FBXL12 synthesis in microglia. **a** Images of spinal cord lesions at different time points after injury; the lesions were stained with antibodies against FBXL12 (green) and IBA1 (red) or with DAPI (blue). **b** Quantification of IBA1- and IBA1/FBXL12-positive cells within the 500 µm range at different time points after injury. (one-way ANOVA, mean ± SEM; *, IBA1 positive, **P < 0.01, ****P < 0.0001, *n* = 5; #, IBA1/FBXL12 positive, ^#^*P* < 0.05, ^####^*P* < 0.0001, *n* = 5; all groups compared with the Sham group). **c**, **d** Immunoblotting of wild-type microglia treated with MBP and LPS as indicated. The graph below shows the blots normalized to β-actin (mean ± SEM, *n* = 3). **e** Immunoblotting of wild-type microglia treated with 200 ng/mL MBP for the indicated times. The graph below shows the blots normalized to β-actin (one-way ANOVA, mean ± SEM; ***P < 0.001, ****P < 0.0001, *n* = 3). **f** RT‒qPCR quantitation of Fbxl12 mRNA expression in SIM-A9 cells treated with 200 ng/mL MBP for the indicated times. mRNA expression is relative to that of the control group (mean ± SEM, *n* = 3). **g** MeRIP‒qPCR quantitation of Fbxl12 mRNA m6A levels in SIM-A9 cells treated with 200 ng/mL MBP for the indicated times. The m6A modification level is relative to that of the control group (*t* test, mean ± SEM; **P < 0.01, *n* = 3). **h** Immunoblotting of FBXL2 expression in microglia with METTL3, METTL14 and YTHDF1 knockdown as indicated. **i** Images of spinal sections at 28 dpi from different groups stained with antibodies against IBA1 and Mettl3 or with DAPI. **j** Images of spinal sections at 28 dpi from different groups stained with antibodies against IBA1 and Fbxl12 or with DAPI. **k** Quantification of IBA1 (left) and IBA1/Mettl3 (right) positive cells at 28 dpi (one-way ANOVA, mean ± SEM; ****P < 0.0001, *n* = 4–5). **l** Quantification of Fbxl12 immunoreactive intensity around the lesion site (left) and microglia (right) at 28 dpi (*t* test, mean ± SEM; ***P < 0.001, *n* = 6–8)

In contrast to those in normal spinal cord physiology, the interactions between many cell types, such as neurons, microglia, astrocytes, and oligodendrocytes, are disorganized, resulting in a harsh organizational environment after SCI. To further verify the major cell type expressing Fbxl12 in response to SCI, we performed coimmunostaining with the cell type-specific markers NeuN, IBA1, GFAP, and OLIG2 for neurons, microglia/macrophages, astrocytes, and oligodendrocytes, respectively. Immunohistochemistry (IHC) of spinal cord injury tissue revealed that IBA1-positive microglia/macrophages transformed from dendritic (as shown in the sham control in Fig. 2a) to amoeboid morphology and migrated toward the injury site (Fig. 2a). We analyzed the expression of FBXL12 in amoeboid and nonactivated Iba1-positive cells at different distances from the injury site (Supplementary Fig. 2d). The results revealed that 77.3% of the microglia exhibited an amoeboid morphology, and among the activated microglia, 95% were positive for FBXL12 proximal to the injury site (a 300 μm × 300 μm area immediately adjacent to the injury site was confined to the proximal region). Among the resting microglia at the distal end of the injury site, 88.3% were nonactivated, and 9.5% were positive for FBXL12 (a 300 μm × 300 μm area located 1 mm longitudinally from the injury center was confined to the distal region). Interestingly, at 3 days postinjury, a significant number of Fbxl12-positive cells, which may represent blood-derived infiltrating immune cells and fibroblasts, were not Iba1 positive. Furthermore, a significant increase in FBXL12/IBA1 costained cells with the ameboid phenotype at 3 days postinjury (Fig. 2b) and in FBXL12/GFAP costained cells at 14 days postinjury was observed; however, no FBXL12/NeuN costained cells were observed, and only a few FBXL12/OLIG2 costained cells appeared at 3 days postinjury (Supplementary Fig. 2a, b). To discriminate resident microglia from infiltrating macrophages during the acute and subacute SCI phases, we used IBA1/TMEM119 coexpression as a marker for microglia. The results demonstrated that the majority of IBA1-positive cells at the injury epicenter maintained TMEM119 expression at 3 and 7 days postinjury (Supplementary Fig. 2c). These data suggest that SCI triggers FBXL12 upregulation mainly in activated microglia surrounding the lesion site.

To determine whether injury stimulates Fbxl12 alteration in microglia, we treated microglia (the SIM-A9 cell line) with lipopolysaccharide (LPS) and myelin basic protein (MBP) in vitro. Interestingly, immunoblotting data revealed that MBP upregulated Fbxl12 expression but that LPS did not upregulate Fbxl12 expression (Fig. 2c, d), which suggested that the TLR4–NF-κB pathway may not be the primary factor involved in FBXL12 upregulation. MBP stimulation (200 ng/mL) resulted in a significant increase in the protein level of FBXL12 on day 4 (Fig. 2e). The CCK8 data revealed that treatment with MBP (200 ng/mL) did not significantly affect microglial cell viability, even after chronic exposure (7 days) (Supplementary Fig. 3a). As a control, MBP stimulation failed to significantly increase the protein level of FBXL12 in astrocytes on day 4 (Supplementary Fig. 3b). However, the qPCR data revealed a slight decrease in the Fbxl12 mRNA level (Fig. 2f). Next, the m6A levels in Fbxl12 mRNA were directly measured via methylated RNA immunoprecipitation, followed by qPCR (MeRIP‒qPCR). This analysis revealed that MBP stimulation significantly increased m6A levels in Fbxl12 mRNA on day 4 (Fig. 2g). These results indicate that m6A modifications may promote FBXL12 protein translation.

Given the functional nature of m6A “writers” and “readers,” to test whether m6A methylation might be involved in FBXL12 translational regulation, we detected FBXL12 expression in “writer”- and “reader”-depleted microglia. The immunoblotting data revealed that the depletion of Mettl3, Mettl14, and YTHDF1 inhibited FBXL12 translation in activated microglia (Fig. 2h and Supplementary Fig. 3c).

To further confirm the regulatory effect of m6A on FBXL12 expression in active microglia, we knocked out Mettle3 in microglia in the spinal cord of mice one week before clamp injury. Immunohistochemistry of spinal cord slices revealed that the number of Mettl3-positive microglia in the AAV-Mettl3-injected group was significantly decreased at 28 days postinjury (Fig. 2I, k). We observed significant downregulation of Fbxl12 expression in microglia after the knockout of Mettl3. Moreover, the injection of AAV-Mettl3 did not alter the number of microglia around the injury site (Fig. 2j, l). These findings further confirm the regulatory role of m6A methylation in the expression of Fbxl12 within activated microglia. Taken together, these data suggest that m6A modification promotes FBXL12 translation in activated microglia.

### FBXL12 regulates cytoskeletal reorganization, promotes its migration and regulates the immune response of microglia

Given the high expression of FBXL12 in activated microglia, we investigated its function in vitro by targeting FBXL12 expression and ablating it in microglia. To further analyze the substrate-binding site of FBXL12 as an E3 ligase, we generated a mutant carrying a mutation in the threonine residue (T128A), which was predicted by RMBase (Supplementary Fig. 3d). Targeted expression and ablation of wild-type and mutant FBXL12 were detected by fluorescence photography, immunoblotting, and qPCR, respectively (Supplementary Fig. 3e, f).

**Fig. 3:**
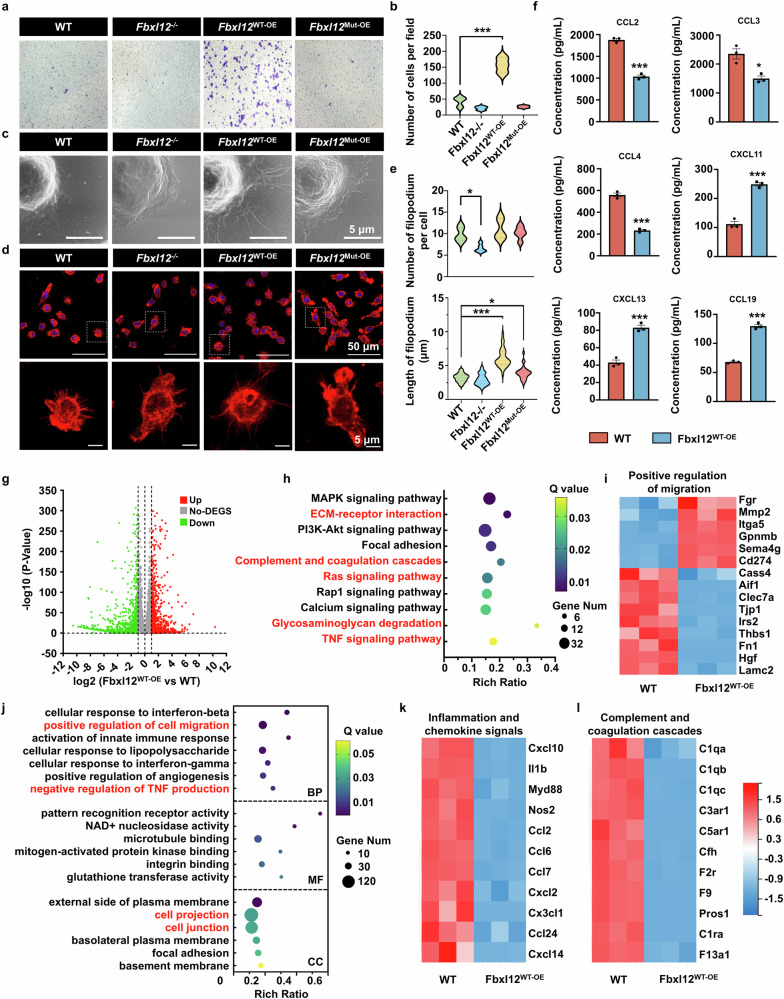
FBXL12 regulates cytoskeletal reorganization, promotes migration and regulates the immune response of microglia. **a** Images of different microglia stained with crystal violet. Where indicated, the cells were wild-type (WT), Fbxl12-overexpressing (Fbxl12^WT-OE^), Fbxl12-mutant (Fbxl12^Mut-OE^) and Fbxl2-deficient (Fbxl12^-/-^). **b** Quantification of migrated microglia in (**a**) (one-way ANOVA, ***P < 0.001, *n* = 3). **c** SEM images of different microglia. **d** Images of different microglia stained with F-actin and DAPI. Representative microglia are shown below the magnified window. Scale bar as indicated. **e** Quantification of filopodium numbers (top, *n* = 5) and lengths (bottom, *n* = 30) of microglia in (**d**) (one-way ANOVA, *P < 0.05, ***P < 0.001). **f** Chemokine secretion in microglia (Fbxl12 ^WT-OE^ vs WT). (*t* test, mean ± SEM; *P < 0.05, ***P < 0.001, *n* = 3). **g** Volcano plot of differentially expressed genes in microglia (Fbxl12^WT-OE^ vs WT). **h** Selected KEGG pathways differentially enriched in WT versus Fbxl12^WT-OE^ microglia. **i** Heatmap of differentially expressed genes associated with positive regulation of the migration pathway in microglia (Fbxl12 ^WT-OE^ vs WT). **j** Gene Ontology (GO) terms enriched in microglia (Fbxl12^WT-OE^ vs WT) were selected. **k**, **l** Heatmap of differentially expressed genes associated with inflammation and chemokine terms and complement and coagulation cascades (**l**) in microglia (Fbxl12^WT-OE^ vs WT)

Because amoeboid-shaped microglia are assumed to actively migrate,^14^ this high motility is closely associated with cytoskeletal reorganization^30^; therefore, we posited that FBXL12 might facilitate microglial migration, which is important for initiating cellular contact between microglia and other glial cells, a prerequisite for injury healing. Transwell and live-cell imaging data revealed that targeting FBXL12 significantly increased SIM-A9 microglial motility, and FBXL12 ablation significantly reduced it. However, mutant FBXL12 overexpression did not affect microglial motility (Fig. 3a, b, Supplementary Videos 1–4, and Supplementary Fig. 3g). Primary microglia from postnatal day 2 (P2) mice edited with AAV were also used to analyze the effect of FBXL12 on microglial migration, and the results were consistent with those from the SIM-A9 microglial cell line. (Supplementary Fig. 3h, i).

Then, we investigated morphological alterations in microglia caused by FBXL12 expression and ablation. Strikingly, scanning electron microscopy images of microglia revealed significant morphological alterations in filopodia. Microglia overexpressing Fbxl12 appear more three-dimensional and plumper, with a significant increase in filopodia, whereas cells with Fbxl12 knockout appear notably flattened (Fig. 3c). Compared with wild-type microglia, ablation of FBXL12 caused a significant decrease in the number of filopodia (Fig. 3d, e). Furthermore, targeting the expression of FBXL12 and mutant FBXL12 significantly increased invasion of filopodia. Consistent with these findings, in primary microglia after FBXL12 deletion, cytoskeleton morphology was distinctly disrupted compared with that in the wild-type group. Unfortunately, we did not observe significant changes related to the filopodia detected in SIM-A9 microglia in primary microglia (Supplementary Fig. 3j). These results suggest that FBXL12 is involved in cytoskeletal reorganization in microglia.

Given that FBXL12 regulates the cell cycle,^26^ we examined the effects of FBXL12 expression and ablation on microglial proliferation. Cell counting and colony formation data revealed that targeting FBXL12 expression significantly inhibited cell growth; however, ablation of FBXL12 had no effect on microglial growth (Supplementary Fig. 3k, l). Moreover, cell cycle analysis revealed that targeted expression of FBXL12 increased the proportion of cells in the G0/G1 and G2/M phases but decreased the proportion of cells in the S phase; however, ablation of FBXL12 increased the proportion of cells in the S phase but decreased the proportion of cells in the G2/M phase (Supplementary Fig. 3m). Notably, targeting the expression of mutant FBXL12 had no effect on proliferation or cell cycle progression, which suggested that the FBXL12 T128A mutant harbors a point mutation leading to a single amino acid change that clearly abrogates its function. Furthermore, we analyzed the phagocytic capacity of microglia overexpressing Fbxl12, and the results revealed that Fbxl12 overexpression did not significantly alter the phagocytic capacity of microglia (Supplementary Fig. 3n, o). Overall, these data indicate that FBXL12 regulates cytoskeletal reorganization and promotes the migration of microglia but inhibits their proliferation.

Microglia are the resident immune cells of the central nervous system that play a role in monitoring the microenvironment. They secrete chemokines to recruit peripheral immune cells and regulate the immune microenvironment in response to injury or pathogen invasion. Therefore, we also examined the impact of Fbxl12 on the chemotactic function of microglia. The results indicated that the overexpression of Fbxl12 led to the downregulation of inflammatory and fibrosis-related chemokines^31–33^ (such as CCL2, CCL3, and CCL4) and the upregulation of homeostatic-related chemokines such as CCL19, CXCL11, and CXCL13 (Fig. 3f).

We conducted RNA-Seq on wild-type and Fbxl12-overexpressing microglia to further elucidate the regulatory role of Fbxl12 in microglia. Differential gene expression is shown in Fig. 3g. Kyoto Encyclopedia of Genes and Genomes (KEGG) analysis revealed that the differentially expressed genes could be enriched in pathways such as the ECM‒receptor interaction, complement and coagulation cascades, the Ras signaling pathway, glycosaminoglycan degradation, and the TNF signaling pathway (Fig. 3h). Similarly, GO analysis revealed enriched terms related to positive regulation of cell migration, negative regulation of TNF production, cell projection, and cell junctions, among others (Fig. 3j). These results confirm our findings regarding the regulation of microglial migration and chemokine secretion by Fbxl12. The expression heatmaps of genes associated with inflammation and chemokine signaling, as well as those associated with the complement and coagulation cascade pathways, are shown in Fig. 3i, k, l.

Interestingly, pathways related to ECM‒receptor interactions and glycosaminoglycan degradation, both of which are associated with scar formation following SCI, were also observed in our KEGG results. Furthermore, a recent study suggested that the inhibition of macrophage/microglia migration after injury disrupted inflammatory diffusion, which increased the scar area.^14^

### Targeting FBXL12 upregulation promotes the migration of microglia after SCI

**Fig. 4:**
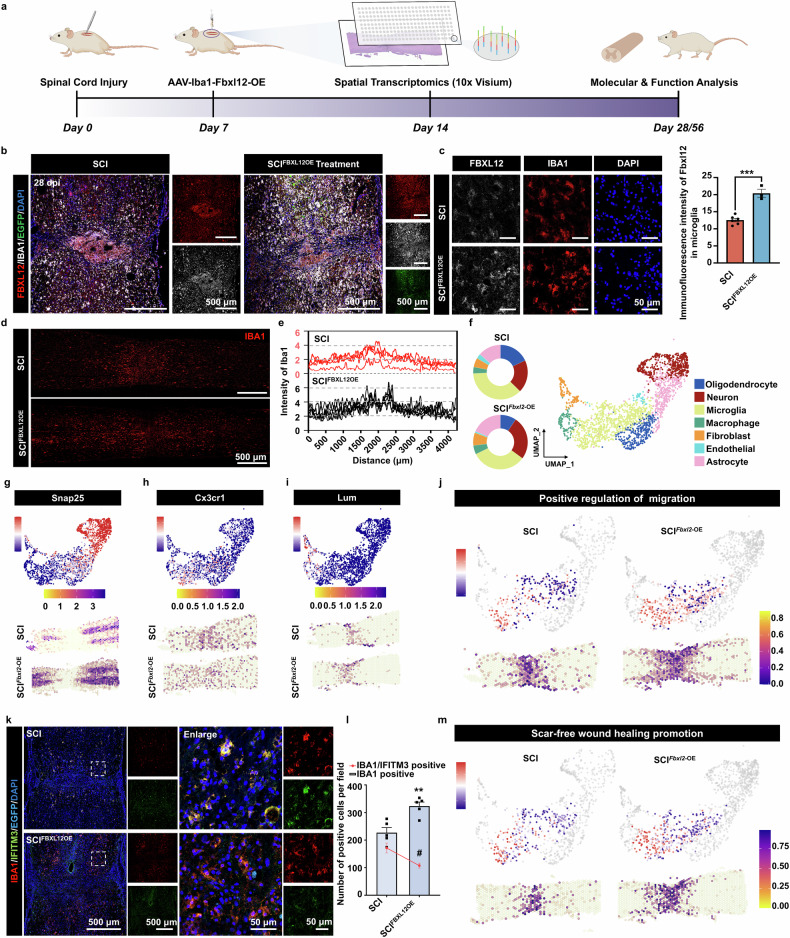
AAV-Fbxl12 delivery promotes microglial migration after SCI. **a** Schematic description of the intrathecal delivery of AAV-Fbxl12. **b** Immunofluorescence staining of spinal cord tissue for FBXL12 and IBA1 at 28 dpi. **c** Images of spinal cord lesions stained with antibodies against IBA1 and Fbxl12 or with DAPI (blue) and quantification of Fbxl12 immunoreactive intensity in microglia at 28 dpi (*t* test, mean ± SEM; ***P < 0.001, *n* = 3–6). **d** Images of the spinal cord stained with IBA1 (4 mm in length centered around the injury site). **e** Quantification of IBA1 immunoreactive intensity (*n* = 5). **f** Uniform manifold approximation and projection (UMAP) plot of spots from all sections visualized via the Seurat package. Through unsupervised clustering and marker gene expression, 7 cell types were annotated. The proportions of various cell types in the SCI and SCI^*Fbxl12*-OE^ groups are presented in pie charts (left). **g–i** Expression profiles of marker genes of neurons, microglia and fibroblasts are presented via UMAP, where each dot represents an individual spot and the color represents the expression level (dark red, high expression; dark blue, low). Spatial distribution of the expression profiles of marker genes in spinal cord sections, with colors representing expression levels (dark blue, high expression; dark blue, low expression). **j** UMAP plot of microglia with other cell types denoted by gray (top) and spatial distribution (bottom) of GSVA scores for positive regulation of migration. **k** Images of spinal sections at 28 dpi stained with antibodies against IBA1 and IFITM3 (*n* = 5). **l** Quantification of IBA1- and IBA1/IFITM3-positive cells within the 1500 µm range at different time points after injury. (*t* test, mean ± SEM; *, IBA1 positive, **P < 0.01, *n* = 5; #, IBA1/IFITM3 positive, ^#^*P* < 0.05, *n* = 5). **m** UMAP plot of microglia with other cell types denoted by gray (up) and spatial distribution (down) of GSVA scores for scar-free wound healing promotion

We reasoned that if microglia are critical in the regulation of the SCI microenvironment, the exogenously promoted mobility of microglia should alter the pathology at the injury site. To avoid the low transfection efficiency of viral injection during the acute phase and to align with existing clinical surgical procedures for spinal cord injury treatment, we injected AAV-FBXL12 (with IBA1 as a promoter and EGFP coexpression) into the spinal cord adjacent to the injury site at 7 days postinjury (Fig. 4a). Viral transduction resulted in the upregulation of FBXL12 in microglia (Fig. 4b, c), and microglia exhibited a more dispersed distribution within the spinal cord (Fig. 4d, e). Additionally, we injected AAV-IBA1-EGFP (with IBA1 as a promoter, EGFP coexpression, or empty plasmid AAV) into the spinal cord adjacent to the injury site at 7 days postinjury as the negative control group (NC group). Compared with that in the SCI group, the microglial distribution in the NC group did not obviously differ (Fig. 4d, e, Supplementary Fig. 4a). Then, spatial transcriptomic sequencing of the spinal cords of mice at 14 days post-SCI was performed to further elucidate the potential mechanisms by which targeted overexpression of Fbxl12 in microglia promotes injury repair. According to the reported marker list, the 10×Visium dataset was visualized via molecular-based classification (Fig. 4f). Compared with that in the SCI group, the proportion of neurons in the AAV-Fbxl12-injected group was significantly greater, as shown in the UMAP graphs of various cell types. *Snap25*, *Cx3cr1* and *Lum* were used as markers of neurons, microglia and fibroblasts, respectively, and the UMAP and spatial positions are presented (Fig. 4g–i). Subsequent GO analysis revealed that the AAV-Fbxl12-injected group presented significantly greater scores for positive regulation of migration (*Fgr, Mmp2, Itga5, Gpnmb, Sema4g*, and *Cd274*) and was affected mainly by microglia (Fig. 4j).

Recently, studies have shown that microglia from postnatal mice can significantly improve functional recovery after SCI, which indicates that the aging state of microglia may be vital to their effect on neural repair.^7^ To further confirm the molecular signatures of microglia with Fbxl12 upregulation, the expression of fatty acid binding protein 5 (FABP5) and interferon-induced transmembrane protein 3 (IFITM3) in the spinal cord was tested. Recent studies utilizing single-cell RNA sequencing have demonstrated that FABP5 is exclusively expressed in the microglia of embryonic mice, whereas IFITM3 is significantly upregulated in microglia during aging and in various neurodegenerative conditions.^34,35^ Immunofluorescence staining of spinal cord sections at 28 days postinjury revealed that AAV-FBXL12 delivery significantly altered FABP5 expression (Supplementary Fig. 4b) and inhibited IFITM3 expression in microglia surrounding the injury center (Fig. 4k, l). Similar results were observed in cellular experiments with IFITM3; however, FABP5 was not upregulated following FBXL12 overexpression in the cellular experiments (Supplementary Fig. 4c). Additionally, compared with those in the SCI group, microglia with upregulated FBXL12 presented scarless wound healing properties (*Ms4a7*, *Thbs1*, *Ms4a6c*, and *Lgals1*), which was reported in the study of Li et al. (Fig. 4m). These results indicate that the upregulation of FBXL12 can enhance the motility of microglia, endowing them with certain wound healing characteristics and reducing IFITM3-dependent senescence-like molecular features.

### AAV-FBXL12 treatment reduces scarring and promotes functional recovery after injury

**Fig. 5:**
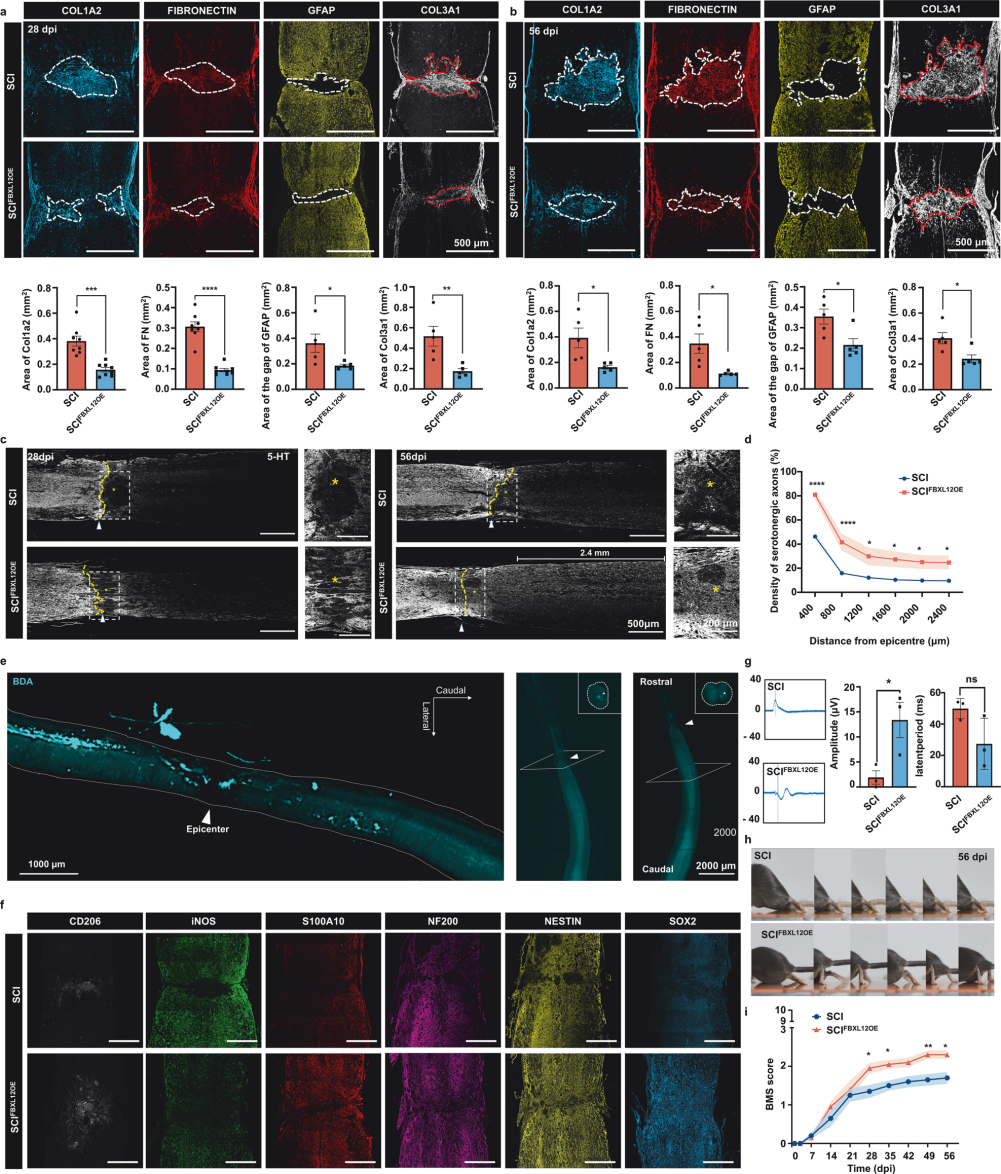
Intrathecal delivery of FBXL12 to microglia reduces scarring, ameliorates pathology and improves axon regeneration in mice. **a**, **b** Images of spinal sections at 28 and 56 dpi stained with antibodies against the indicated proteins. Quantification of the indicated immunoreactive area in the lesion site below the images (dashed area in images) (*t* test, mean ± SEM; *P < 0.05, **P < 0.01, ***P < 0.001, ****P < 0.0001, *n* = 5–8). **c** Images of anti-5-HT-stained spinal sections from different groups of mice at 28 dpi and 56 dpi, showing serotonergic axons. Yellow stars indicate the lesion site. **d** Quantification of the density of serotonergic axons (normalized to the density proximal to the lesion site) in the spinal cord distal to the lesion site at 56 dpi (two-way ANOVA, mean ± SEM; *P < 0.05, ****P < 0.0001, *n* = 5–7). **e** Orthogonal projection image of the spinal cord with tissue clearing and 3D imaging. **f** Images of spinal sections at 28 dpi stained with antibodies against CD206, iNOS, S100A10, NF200, NESTIN and SOX2. **g** Amplitude and latency period of MEPs at 56 days postinjury (*t* test, mean ± SEM; *P < 0.05, *n* = 3). **h** Representative images of hindlimb movement in mice at 56 dpi with or without AAV-FBXL12 overexpression. **i** BMSs of mice with or without AAV-FBXL12 overexpression (two-way ANOVA, mean ± SEM; *P < 0.05, **P < 0.01, *n* = 10)

The upregulation of FBXL12 in microglia confers a series of characteristics that are beneficial for injured spinal cord repair. Thus, we verified the favorable outcomes associated with targeting FBXL12 in microglia following SCI. Following AAV-FBXL12 treatment, a significant decrease in the COL1A2-defined scar area was observed, with an average 58.9% reduction at 28 dpi and a 58.6% reduction at 56 dpi (Fig. 5a, b). Notably, mice in the group that was administered AAV-FBXL12 at 7 days postinjury presented significant reductions in scar areas of 69.3% and 67.4%, as indicated by the FIBRONECTIN at 28 and 56 dpi, respectively (Fig. 5a, b). We subsequently examined the GFAP-deficient area and COL3A1 expression at the injury site after SCI to characterize scar formation further. The results indicated that AAV-FBXL12 delivery significantly reduced the GFAP gap by 48.8% and 39.2% at 28 and 56 dpi, respectively, and COL3A1 expression by 66.3% and 39.9% at the injury site at 28 and 56 dpi, respectively (Fig. 5a, b). The expression of chondroitin sulfate proteoglycans (CSPGs), another marker of scar formation, was found to decrease following AAV-FBXL12 delivery, with a significant reduction observed at 56 days postinjury (Supplementary Fig. 4d). Furthermore, the results from Masson’s trichrome and Sirius Red staining also demonstrated a reduction in scar formation (Supplementary Fig. 4e). These data indicate that AAV-FBXL12 injection at 7 days postinjury resulted in scarless wound healing.

Subsequent GO analysis of the 10×Visium dataset revealed that more ECM receptor interactions occurred at the injury site in the AAV-FBXL12 delivery group (Supplementary Fig. 4f), and these alterations regulated the formation and development of scar tissue, potentially contributing to the reduction in the scar area. Importantly, compared with that in the SCI group, neural crest formation, which occurred primarily in clusters of neurons, was significantly greater after AAV-Fbxl12 delivery (Supplementary Fig. 4g). To present the cell components more clearly, the proportions of each cell type detected within a single spot are shown as a pie chart, and the entire spinal cord section is displayed as an assembly of spatial pie charts. The spots in the injury center presented greater proportions of neurons, oligodendrocytes and astrocytes and a lower proportion of macrophages (Supplementary Fig. 4h).

The scar is a key spatial barrier that inhibits axonal regeneration; therefore, we investigated whether scarless repair induced by FBXL12 could promote axonal regeneration. Invigoratingly, AAV-FBXL12 delivery resulted in more serotonergic axons crossing the lesion center 28 days postinjury, and regeneration of serotonergic axons was detected at a length greater than 2.4 mm 56 days postinjury, in contrast to the SCI group (Fig. 5c). The intensity of 5-HT below the injury site was significantly greater in the AAV-FBXL12 delivery group than in the SCI group, especially in the area around the injury core (Fig. 5d). The downregulation of Fbxl12 in microglia after SCI did not result in significant changes in the area or density of scars, and there was no improvement in the expression of 5-HT (Supplementary Fig. 5a–d). The growth of corticospinal axons was also analyzed on the basis of biotinylated dextran amine (BDA)-labeled axons. AAV-FBXL12 delivery resulted in CST axons growing and crossing through the injury center scar tissue at 56 days postinjury (Fig. 5e and Supplementary Videos 5 and 6). Additionally, we analyzed axon morphology through Tuj1 staining in the injury center and observed Tuj1-positive filamentous fluorescence in the injury center after AAV-FBXL12 delivery; however, this phenomenon was not observed in the SCI group (Supplementary Fig. 5e). We also investigated the inflammatory response and the phenotype of astrocytes at the injury site to evaluate the immune environment after injury. The data revealed significant downregulation of the proinflammatory gene iNOS following AAV-Fbxl12 injection. Additionally, astrocytes tended toward an A2 homeostatic phenotype identified as S100A10, and microglia tended toward an M2 anti-inflammatory phenotype identified as CD206 (Fig. 5f). On the basis of the promotion of healing by AAV-FBXL12 delivery at 7 days postinjury, we further assessed the expression of stemness- and axon-related genes around the lesion site at 28 days postinjury. There was a clear gap between the two stumps of the clamped spinal cord, and neurofilaments and NESTIN^+^ cells stopped rostral to the lesion, whereas SOX2^+^ cells were absent around the injury site. Notably, in AAV-FBXL12-treated mice, the gap was filled with NF200^+^, NESTIN^+^, and SOX2^+^ cells.

Furthermore, electrophysiological analysis was applied to assess the recovery of neural conduction following AAV-FBXL12 delivery. Compared with that in the SCI group, the amplitude of the electrophysiological signals was significantly greater, and the latency was significantly shorter after AAV-FBXL12 delivery, suggesting that signal transduction was restored to some extent^8^ (Fig. 5g). Additionally, limb function was evaluated via the Basso Mouse Scale (BMS). In the AAV-FBXL12 delivery group, the bilateral hind limbs of most of the mice began to show movement exceeding half of the ankle joint excursion by 28 days postinjury. At 49 days postinjury, some mice demonstrated dorsal stepping in one hind limb. Unfortunately, occasional plantar stepping was not observed. In contrast, in the SCI group, only a single hind limb or a very small number of bilateral hind limbs exhibited movement exceeding half of the ankle joint excursion (Fig. 5h, i and Supplementary Videos 7–9).

Moreover, UMAP and the spatial position of complement and coagulation cascade terms revealed that complement secretion at the injury site was significantly decreased after AAV-FBXL12 treatment, and this phenomenon was highly consistent with the RNA-Seq results indicating that microglia overexpress Fbxl12 in vitro. (Supplementary Fig. 5f and Fig. 3l). In addition, the distance between NeuN-positive neurons at the rostral and caudal ends of the injury center was also analyzed. The results showed that AAV-FBXL12 delivery shortened the distance between NeuN-positive neurons separated by the injury center (Supplementary Fig. 5g). These results suggest that after SCI, microglia exhibit enhanced motility after AAV-Fbxl12 injection, reducing chemotaxis toward peripheral monocytes, regulating the immune microenvironment, and mitigating damage from the complement pathway.^36,37^ These data demonstrate that AAV-FBXL12 can facilitate neural cell regeneration and functional reestablishment of the injured spinal cord.

### FBXL12 mediates MYH14 K63 ubiquitination to orchestrate cytoskeletal reorganization in microglia

**Fig. 6:**
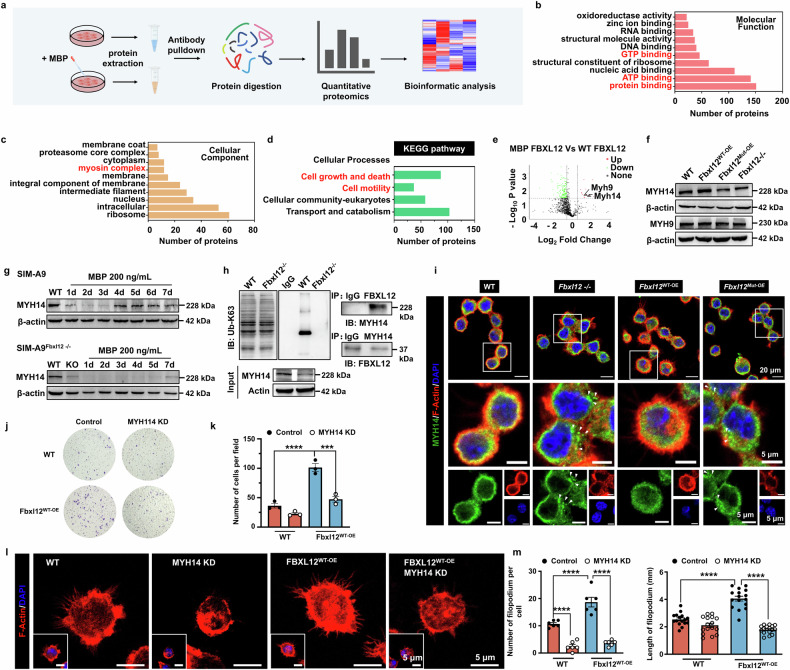
FBXL12 mediates MYH14 K63 ubiquitination to orchestrate the cytoskeletal reorganization of microglia. **a** Schematic description of immunoprecipitation‒mass spectrometry (IP‒MS) of microglia treated with 200 ng/mL MBP for 4 days. **b**, **c** GO-molecular function and cellular component analyses of whole FBXL12-binding proteins. **d** KEGG pathway analysis of MBP-treated microglia. **e** Volcano plot of FBP changes in wild-type microglia and MBP-treated microglia. **f** Immunoblotting of MYH14 and MYH9 in different microglia. **g** Immunoblotting of MYH14 in WT and Fbxl12^−/−^ microglia treated with 200 ng/mL MBP for the indicated times. **h** Immunoprecipitation of FBXL12 with MYH14 in WT and Fbxl12^−/−^ microglia. **i** Images of different microglia stained with F-actin and antibodies against MYH14. Representative microglia are shown in the middle and below magnified windows. Scale bar as indicated. **j** Crystal violet staining of WT and Fbxl12^WT-OE^ microglia. **k** Quantification of migrated microglia in **j** (one-way ANOVA, mean ± SEM; ***P < 0.001, ****P < 0.0001, *n* = 3). **l** Images of WT and Fbxl12^WT-OE^ microglia stained with F-actin, with or without MYH14. **m** Quantification of the number (left, *n* = 6) and length (right, *n* = 16) of filopodium in **l** (one-way ANOVA, mean ± SEM; ****P < 0.0001)

To investigate the substrate by which FBXL12 mediates migration in activated microglia, we performed liquid chromatography‒tandem mass spectrometry (LC‒MS/MS) to compare the FBXL12-binding protein (FBP) profile following microglial stimulation with that of MBP (Fig. 6a). Proteomic analysis revealed significant alterations in FBPs in activated microglia (Supplementary Fig. 5h). Molecular functional analysis revealed that the largest subset of FBPs is involved primarily in “protein binding,” followed by “ATP binding” and “GTP binding” (Fig. 6b), and cellular component analysis revealed that five proteins belong to the myosin complex among the top 10 FBPs with significant changes (Fig. 6c and Supplementary Fig. 5i). Notably, KEGG analysis revealed that the FBPs were involved mainly in “cell growth and death” and “cell motility” and may participate in the “immune system” and “nervous system” (Fig. 6d). These findings imply that FBXL12 substrates may be ATP- or GTP-dependent proteins that mediate cytoskeletal reorganization or cell migration. We identified five myosin proteins among these potential substrates, including three myosin light chain proteins (MYL10, MYL6, and MYL6b) and two myosin heavy chain proteins (MYH9 and MYH14) (Fig. 6e and Supplementary Fig. 5i). These data suggest that myosin is a substrate for FBXL12 in activated microglia.

Given that myosin is a hexamer consisting of several domains and that the heavy chain domains have ATPase activity and interact directly with actin,^38,39^ we first investigated whether FBXL12 regulates MYH9 and MYH14 levels via protein degradation. Unexpectedly, immunoblotting data revealed that the abundance of MYH14, which targets FBXL12-overexpressing microglia, was reduced in microglia with FBXL12 ablation and in those with mutant FBXL12-expressing microglia. However, MYH9 expression did not differ among conditions (Fig. 6f).

Next, we determined whether FBXL12 was necessary for the upregulation of MYH14 in activated microglia. The immunoblotting data revealed that MBP stimulation upregulated MYH14 expression in wild-type microglia, which was consistent with the findings for FBXL12. In contrast, MBP stimulation did not induce MYH14 upregulation in FBXL12-deficient microglia (Fig. 6g). These data suggest that FBXL12 positively regulates MYH14 expression in activated microglia. Coimmunoprecipitation revealed that FBXL12 bound to MYH14 in microglia, which was consistent with the LC‒MS results (Fig. 6h). Given that FBXL12 enhances substrate stability via K63-linked ubiquitination, we performed coimmunoprecipitation to assess the K63-linked ubiquitination of MYH14 in FBXL12-knockout microglia. Immunoblotting data revealed that K63-linked ubiquitination of MYH14 decreased in FBXL12-deficient cells (Fig. 6h). Moreover, fluorescence immunocytochemistry data revealed the colocalization of MYH14 and F-actin in microglia expressing wild-type and FBXL12 (Fig. 6i). Notably, we detected more aggregated particles of MYH14 in FBXL12-ablated and FBXL12-targeting microglia than in wild-type and FBXL12-targeting microglia. Given that the actin cytoskeleton exerts a pulling force through myosin activity, which is a power source for cell migration, myosin has been reported to mediate cytoskeletal reorganization. Next, we examined whether MYH14 is a substrate of FBXL12, which directly mediates cytoskeletal reorganization of microglia. We depleted MYH14 via small interfering RNA (siRNA) in wild-type and FBXL12-expressing microglia (Supplementary Fig. 5j). In addition, Transwell data revealed that MYH14 knockdown effectively blocked the promotion of migration caused by FBXL12 overexpression (Fig. 6j, k). Immunocytochemistry data revealed that the depletion of myosin significantly decreased the number and length of filopodia in microglia (Fig. 6l, m).

## Discussion

During SCI, microglia are closely involved in pathological processes,^8,40^ including immune regulation,^41,42^ cytokine communication,^43^ and scar formation.^44^ In terms of scar formation, recent studies on microglia have received considerable attention and have made several breakthroughs; however, the efficacy of promoting nerve regeneration has yet to be satisfactory. In our work, we applied AAV-FBXL12 to regulate several important aspects of microglial behavior in SCI: (1) Cell motility in response to microglial dispersion and (2) an “antiaging-stable” state characterized by low chemotaxis and reduced complement secretion conducive to recovery from SCI and (3) an improved microenvironment in the injury center for scarless repair. Our results suggest that promoting the entry of an appropriate number of genetically edited microglia into the injury center at the right time point is conducive for scar-free injured spinal cord repair. Future studies should explore the functional behaviors of microglia at lesion sites at different time points and the relative mechanisms of metabolism, migration, and phagocytosis, from which glial and fibrotic scars are removed.

SCI disrupts critical neural pathways that mediate signal transmission from the brain to peripheral effectors through spinal cord circuits. The corticospinal tracts and serotonergic axons, in particular, serve essential functions in transmitting the neural signals required for precise motor coordination.^45^ Consequently, promoting axonal regeneration through spinal lesions has emerged as a promising therapeutic strategy for both pathological repair and functional recovery post-SCI, with significant research efforts dedicated to this therapeutic avenue. Experimental interventions such as PTEN gene deletion and AAV2-hIL-6 delivery have demonstrated regenerative capacities, enabling axonal regrowth of ~1 mm and 7 mm beyond lesion sites, respectively.^46,47^ Notably, this scar-penetrating axonal regeneration correlates with measurable improvements in motor function recovery in SCI animal models. The enhanced regenerative potential of engineered IL-6 has been mechanistically linked to its activation of the JAK/STAT3 intracellular signaling cascade. While substantial advancements have been made in elucidating axonal regeneration mechanisms in the central nervous system over recent decades, clinical translation remains challenging, as evidenced by persistent limitations in neurological recovery following SCI. This therapeutic gap may be partially explained by the persistent presence of residual glial scarring within the injury epicenter.

As reported in previous studies, transplantation of neonatal or adult microglia treated with a peptidase inhibitor improved healing, as shown by the reduction in collagen I and CSPG deposition,^7^ leading to scar-free recovery. These works indicate that the state of microglia determines their role in spinal cord injury, whether for scar formation or repair promotion. This study highlights the importance of microglia in the repair of SCI, suggesting that regulating the state of microglia could be a breakthrough for scarless injured spinal cord repair. However, current efforts to alter the state of microglia lack effective regulatory targets. In our study, we focused on scarless repair in adult SCI model mice and explored the mechanisms and regulatory strategies of scar formation via multiple transcriptomes, including transcriptomic, epitranscriptomic, and posttranslational regulation.

As the most prevalent and abundant epitranscriptomic modification, m6A methylation plays an essential role in pathological processes.^48^ Here, we identified m6A as the main epigenetic mRNA modification involved in SCI progression, which is consistent with previous works.^49^ Through in-depth analysis of multiple transcriptomes, we identified Fbxl12 as a key gene involved in injured spinal cord repair. This protein belongs to the E3 ubiquitin ligase family, and proteins from this family have been reported to be closely associated with the youthfulness and metabolism of stem cells, which is also associated with stem cell youthfulness and metabolism.^50^ m6A-dependent Fbxl12 upregulation indeed alternated with the state of microglia after SCI, including cell cycle arrest, migration promotion and youthful state maintenance. Coincidentally, the results of the scar-less repair of SCI achieved by our engineered antiaging-stable microglia validate the work of Li et al., who used the transplantation of newborn mouse microglia to promote scar-free repair of SCI.^7^ Furthermore, we revealed a novel regulatory target that can modulate the state of microglia in situ after injury, resulting in more significant scarless repair and axonal regeneration. In the future, this regulatory target will undoubtedly be more convenient and feasible for the clinical treatment of SCI.

The scars were significantly diminished, resulting in a significant reduction in the areas of glial and fibrotic scars. Furthermore, serotonergic axons growing through the center of the lesion site at 28 days postinjury, which reached a distance greater than 2.4 mm, were clearly observed compared with those at 56 days postinjury. Compared with the scar-cleaning studies mentioned above, our study investigated the intervention target and time window for scar removal in adults with SCI. AAV-FBXL12 delivered 7 days but not 3 days postinjury promoted functional recovery, which may be explained by the different pathological conditions between these windows. Overall, we identified FBXL12 as a promising target for scar removal via microglia, and 7 days postinjury was the appropriate intervention time window.

The literature indicates that reducing the recruitment of peripheral monocytes promotes spinal cord injury repair. Inhibition of pericyte proliferation reduces fibrotic scar tissue following injury and facilitates motor axon regeneration.^36^ After SCI, microglia recruit peripheral cells by secreting chemotactic factors, such as CCL2, CCL3, and CCL4. However, these chemotactic factors have been reported to contribute to secondary damage after spinal cord injury, possibly because the recruitment of peripheral monocytes exacerbates the inflammatory storm.^31–33,37^ Among the cytokines secreted by immune cells, complement factors may be the main culprits in the activation of astrocytes and neuronal death. Reducing complement system secretion plays a key role in the repair of SCI. These findings indicate that the upregulation of Fbxl12 in microglia reduces the recruitment of peripheral immune cells and complement secretion, which is likely the key reason for the inhibition of astrocyte activation, protection of neurons, and promotion of scar-less repair of SCI and functional recovery in mice after injury. In our study, when Fbxl12 was overexpressed in microglia, significant downregulation of chemotactic factors such as CCL2, CCL3, and CCL4 was observed. The RNA-Seq results indicated that the differentially expressed genes were enriched in downregulated inflammatory and chemotactic pathways, alongside a significant downregulation in the complement pathway. The reduced complement secretion was validated via spatial transcriptomics data, with a significant decrease in complement expression at the SCI site following targeted overexpression of Fbxl12 in microglia. These results suggest that the upregulation of Fbxl12 in microglia reduces the recruitment of peripheral immune cells and complement secretion, thereby protecting neurons and promoting functional recovery in mice following injury.

Owing to the difficulty of achieving satisfactory recovery from regenerated axons, the activation and modulation of endogenous renascent neural stem cells (NSCs) constitute another influential theory. Therefore, whether these endogenous neural stem cells can break through dense scar tissue and form fresh neurons is a critical issue that needs to be addressed. Implanting neural regeneration scaffolds and/or NSCs directly promotes the entry of renascent stem cells across lesion scars and their differentiation into neurons.^40^ However, in addition to surgical transplantation strategies, AAV-FBXL12 delivery reduced the density of the residual scar, and as a result, many NESTIN^+^ and SOX2^+^ cells were observed in the lesion core. Hence, our study revealed the proper regeneration microenvironment for axon regeneration and excitation of endogenous NSCs. These results suggest promising strategies, such as incorporating AAV-FBXL12 with neurotrophin delivery and codelivery of FBXL12 with additional neural regeneration-related genes.

IHC analysis revealed aberrant expression of FBXL12 in microglia with the ameboid phenotype compared with that in the sham group. Morphological phenotypes have traditionally been used to characterize the activated state of microglia, and the highly dynamic nature of microglia in response to injury was first reported in 2005.^51^ In fact, we also found that the accumulation of FBXL12 in astrocytes begins 14 days postinjury, suggesting that the regulation of FBXL12 in astrocytes might occur during the late subacute stage but lasts throughout the pathological process in microglia. In addition, further study on the regulation of FBXL12 in pathological astrocytes is meaningful, as it could contribute to revealing the mechanism of Fbxl12 regulatory function. Fbxl12 is an E3 ligase that targets several proteins to modulate cytophysiological activities.^26,52^ Notably, FBXL12 promoted the expression of the CDK inhibitor p21 by increasing K63-linked ubiquitination and inducing the decay of proliferation in HEK293 cells.^53^ Consistently, our results revealed that FBXL12 mediated the K63-linked ubiquitination of MYH14 and that this ubiquitination promoted the accumulation of MYH14. In addition, we generated a mutant carrying a mutation in the threonine residue (T128A) to preliminarily screen for the substrate-binding site of FBXL12. Interestingly, targeting the expression of this mutant affected either the migration or proliferation of microglia but inhibited colony formation and slightly affected cytoskeletal reorganization. These data imply that threonine is involved in the substrate binding of FBXL12.

We reported that the accumulation of MYH14 regulated by FBXL12 promoted the migration of microglia through cytoskeletal reorganization, resulting in a significant increase in the length of filopodia. Indeed, cell migration is essential for physiological processes such as the immune response, tissue repair, and development.^54,55^ As a highly dynamic process, the roles and functional cooperation of cytoskeletal reorganization and myosin in cell migration have been well described.^39,56^ Amoeboid cells lack focal adhesions and use actin-driven protrusions to glide on substrates much faster than mesenchymal cells do. These amoeboid characteristics might explain the rapid migration of activated microglia toward the injury site, which ultimately leads to the removal of glial scars and fibrotic scars at the SCI lesion site. Additionally, excessive proliferation of microglia is a critical pathological phenomenon after SCI. Therefore, we speculate that targeting FBXL12 in microglia inhibits the excessive proliferation of microglia during the subacute phase of SCI. This may also be a potential reason why AAV-FBXL12 delivery promotes scarless repair of SCI.

FABP5, which is involved in fatty acid metabolism in microglia, is predominantly expressed at E14.5 in postnatal mice.^34^ Unfortunately, no alterations in FABP5 were observed in our study in vitro or in vivo. IFITM3, which reportedly functions in immune activation and is highly expressed in pathological or aging microglia,^35^ was significantly decreased when microglia were present earlier around the injury site. This phenomenon suggests that rapidly arriving microglia may participate in immune regulation earlier, alleviating chronic immune dysfunction. In addition to their appropriate spatial and temporal distribution, the antiaging state of microglia may also be crucial for injury repair.

Indeed, AAV-FBXL12 delivery did not achieve complete scarless repair. This limitation likely arises from the complex involvement of multiple cell types in regulating the postinjury microenvironment following SCI. Targeting microglial activity alone was insufficient to fully reverse the adverse effects on the tissue microenvironment. Future research should prioritize the identification of key regulatory genes in additional cell populations, such as astrocytes, fibroblasts and oligodendrocytes. Recent advancements in multiomics technologies have revealed the crucial regulatory roles that fibroblasts play in the pathophysiology of spinal cord injury. Our current characterization of strongly Fbxl12-positive cell populations remains incomplete, and we acknowledge the strong possibility that fibroblasts may constitute one of these unidentified cellular targets. Our experimental evidence collectively suggests that Fbxl12 may exert regulatory functions through multiple cellular mediators, potentially including astrocytes, neutrophils, and fibroblasts. This multifaceted interaction network warrants further systematic investigation to fully elucidate the molecular mechanisms underlying the role of Fbxl12 in neural injury responses.

In addition, inhibiting scar formation creates a permissive microenvironment that facilitates the regeneration of serotonergic neurons and corticospinal tract axons through the glial scar barrier. Unfortunately, the observed 2.4 mm regenerative growth triggered by AAV-FBXL12 delivery fails to achieve the necessary path length for regenerated axons to establish functional connectivity with their distal target regions. These findings therefore suggest that combinatorial therapeutic strategies— simultaneously addressing axonal regeneration through enhancing the intrinsic growth capacity and scar-free repair—should be prioritized in future spinal cord injury research to bridge this critical translational gap.

Taken together, our results suggest that targeting FBXL12 in microglia 7 days postinjury could provide a novel method to effectively diminish scar formation triggered by SCI and promote effective axon regeneration through the injury center. On the basis of our findings of scarless wound healing and an improved microenvironment, future combinatorial interventions with neurotrophins or other clinical treatments could maximize functional recovery after SCI, potentially in humans.

## Materials and methods

### Animals

All surgical and experimental procedures involving rodents were approved and performed in accordance with the standards of the Animal Welfare Committee of Tongji University in Shanghai, China. The animals were housed in groups of six in a pathogen-free barrier facility in corn bedding-lined cages with pellet chow and water bottles.

### SCI model

Eight-week-old C57BL/6 mice weighing 18–20 g were used for our SCI studies. The selected C57BL/6 mice were evenly divided into males and females and were randomly assigned to the required experimental groups. The mice were anesthetized with inhaled isoflurane (2%) delivered in oxygen-enriched air. All animal operations were performed under a dissecting microscope (Nikon/Zeiss) and a rodent stereotaxic apparatus (David Kopf). Laminectomy was performed as previously described. Briefly, the lamina at T9 was removed to expose the spinal cord. No.5 Dumont forceps (Fine Science Tools) fixed on a stereotaxic apparatus were used to clamp the spinal cord for 3 s. For the sham group, laminectomy was performed on the T9 lamina without subsequent clamp injury. The bladders were squeezed to assist micturition, and the animals were monitored once daily to avoid infection. All in vivo experiments were performed by the same experimenter who was blinded to the experimental groups.

### Spinal tissue processing

The animals were perfused with ice-cold 4% paraformaldehyde (PFA; Sigma) in phosphate-buffered saline (pH 7.4; Sigma) after excessive inhalation of isoflurane. Spinal cord tissue was dissected and placed in 4% PFA at 4 °C overnight, then removed to 30% sucrose, and placed overnight at 4 °C for 48 h. After complete dehydration, a 1 cm tissue segment centered around the injury site was encapsulated in Tissue-Tek O.C.T. (OCT) on dry ice. Sample sagittal sections were collected at a thickness of 10 μm with a cryostat (Leica), placed on glass slides and stored at −80 °C. For tissue clearing, spinal cord samples were processed through sequential dehydration in methanol/water gradients (20%, 40%, 60%, 80%, and 100% methanol) with 1 h incubation per concentration at room temperature (RT), followed by overnight incubation in 66% dichloromethane (DCM)/33% methanol under continuous agitation. Rehydration was performed through reverse methanol gradients (80%, 60%, 40%, 20%) and PBS for 1 h per concentration.

### RNA extraction, transcriptome-seq, epitranscriptomic microarray and LC‒MS-based mRNA modification analysis

The spinal cords were divided into different groups according to the number of days postinjury (sham, 15 min, 1, 3, 7, 14, 28, and 42 days). Each group was composed of three replicates. The 1 cm long spinal cord segments centered on the injury site were homogenized in 1 mL of TRIzol (Invitrogen), and total RNA was extracted and purified via a RNeasy Mini Kit (QIAGEN) according to the manufacturer’s instructions. The RNA quality and concentration were measured via a spectrophotometer (Thermo Fisher Scientific). Transcriptome sequencing, epitranscriptomic microarray and LC‒MS-based mRNA modification analyses were conducted at the Shanghai Kangcheng Biotechnology Co., Ltd.

### Protein immunoblotting

For immunoblotting of spinal cord tissue, 1 cm long spinal cord segments centered on the injury site were lysed in 50 mM Tris (pH 7.4), 150 mM NaCl, 1% Triton X-100, 1% sodium deoxycholate, 0.1% SDS, 2 mM sodium pyrophosphate, 25 mM β-glycerophosphate, 1 mM EDTA, 1 mM Na_3_VO_4_, 0.5 μg/mL leupeptin, complete protease inhibitor cocktail (Roche), and 1 mM PMSF (Sigma‒Aldrich). For immunoblotting of whole-cell lysates, the cells were collected in PBS and lysed in 50 mM Tris (pH 7.4), 150 mM NaCl, 1% NP-40, 0.5% sodium deoxycholate, complete protease inhibitor cocktail (Roche), and 1 mM PMSF (Sigma-Aldrich). Protein was quantified via a BCA protein assay kit (Pierce). For immunoprecipitation, whole-cell lysates were incubated with an antibody overnight at 4 °C, followed by incubation with Dynabeads (Thermo Fisher) for 3 h at 4 °C. Equal amounts of proteins from all conditions were loaded onto a 10% SDS‒PAGE gel and transferred onto a nitrocellulose membrane (Millipore). The membrane was blocked with 5% milk for 1 h at room temperature and incubated with primary antibodies diluted with 5% BSA overnight at 4 °C, followed by incubation with secondary antibodies on the second day. After washing, the membrane was incubated with an ultrahigh-sensitivity ECL kit (MCE) and visualized via a chemiluminescence scanner (Tanon). The antibodies used in this study are listed in Supplementary Table 1.

### qRT‒PCR

For qRT‒PCR, cDNA synthesis was performed via the PrimeScript PT Kit (TaKaRa Bio), followed by quantitative PCR of a 10 µL reaction mixture (5 µL of whole-cell lysate TB Green, 0.2 µL of primer, 0.2 µL of Rox reference dya, 1 µL of cDNA, and 3.6 µL of ddH_2_O) with TB Green Premix EX Taq (TaKaRa Bio). The qPCR primers used are listed in Supplementary Table 2.

### Immunohistochemistry and immunocytochemistry

For immunofluorescence analysis, sagittal sections of the spinal cord were blocked with 5% BSA and 0.3% Triton X-100 for 1 h, followed by incubation with primary antibodies at 4 °C overnight. The next day, the slides were washed with TBST and incubated with Alexa Fluor-conjugated secondary antibodies for 1 h at room temperature. All the slides were mounted with Vectashield anti-fade medium.

For immunocytochemistry of cultured cells, the cells were fixed with 4% PFA for 20 min at room temperature, followed by washing with PBS three times. The cells were then treated with 0.25% Triton X-100 for 10 min and washed once with PBS. Next, the cells were incubated with primary antibodies at 4 °C overnight, followed by incubation with Alexa Fluor-conjugated secondary antibodies on the second day. All the cell samples were mounted in VECTASHIELD antifade medium. The images were captured via a Zeiss microscope (LSM880). The antibodies used in this study are listed in Supplementary Table 1.

For paraffin section staining, the tissue was fixed in 10% formalin fixative solution and embedded via routine dehydration. The slices were cut into 4 μm pieces. The samples were dewaxed with water. For Masson’s trichrome staining, the tissue sections were initially stained with hematoxylin for 5 min, followed by rinsing with distilled water. Acidic differentiation solution was applied for 30 seconds, and the sections were blued by rinsing under running tap water for 10 min and then briefly washed with distilled water. The sections were subsequently stained with Ponceau-acid fuchsin solution for 10 min, briefly rinsed with distilled water (<10 seconds), and differentiated with phosphomolybdic acid solution for 2 min. Next, light green staining solution was applied for 1 min, followed by brief rinses with distilled water and, finally, differentiation via acidic differentiation solution for 1 min. Dehydration was achieved through a graded ethanol series, followed by xylene clearing. The sections were permanently mounted with neutral balsam for microscopic examination. For Sirius red staining, tissue sections were stained with Sirius Red Staining Solution for 10 min. The mixture was rinsed quickly with distilled water to remove the excess solution. The samples were quickly dehydrated with a series of ethanol solutions, cleared with xylene, and finally sealed with neutral balsam for microscopic examination.

For immunolabeling, the tissues were blocked overnight in PBS containing 0.2% Triton X-100, 10% DMSO, and 5% normal donkey serum. Primary antibody incubation (1:500 dilution) was conducted for 48 h in PBS supplemented with 0.1% Tween-20, 10 μg/ml heparin, and 5% normal donkey serum. The samples were subsequently washed with PBS/0.1% Tween-20/heparin buffer for 12 h with buffer replacement every 2 h. Secondary staining with Alexa Fluor-conjugated antibodies (1:500; Thermo Fisher Scientific) was performed for 48 h in the same buffer, followed by 24 h of washing with buffer changes every 6 h. All the incubations were performed under gentle rotation. The final clearing process involved methanol gradient dehydration (20%, 40%, 60%, 80%, and two 100% steps), 3 h of treatment with 66% DCM/33% methanol, two 15-min washes in 100% DCM, and immersion in dibenzyl ether (DBE). The tubes were inverted prior to imaging to ensure solution homogeneity. The sample and image solution were mounted on a 10 cm×6 cm×5 cm imaging chamber and imaged on a lightsheet microscope (Fslight, Qihao bio, Inc.). The sample was imaged at 5× magnification, with a xy pixel size of 2.6 μm × 2.6 μm and a step size of 2.6 μm in the z direction.

### Axon quantification

To quantify the regeneration of CST axons after SCI, sagittal sections through the lesion were stained with antibodies against 5-HT. A series of rectangular segments 400 μm wide and 400 μm long covering the dorsal–ventral aspect of the cord was superimposed onto the sagittal sections, starting from the lesion center up to a defined distance caudal. After the background was subtracted, the pixel value of each segment was normalized by dividing by the rostral segment (1 mm rostral). The results are presented as ratios at different distances (axon density indices).

### CST tracing

To label corticospinal tract (CST) axons via anterograde tracing, we administered a total volume of 2.0 μL of BDA (10%, 10,000 MW, Invitrogen D1956) into the sensorimotor cortex at four distinct sites (coordinates relative to bregma in mm: anterior-posterior/medial-lateral/dorsal-ventral, 1.0/1.5/0.6, 0.5/1.5/0.6, −0.5/1.5/0.6, −0.5/1.5/0.6, and −1.0/1.5/0.6). Each injection site received 500 nL of BDA at a rate of 100 nL/min. After each injection, the needle was left in place for 5 min to ensure adequate diffusion of the BDA solution before moving to the next site. The surgical wound was then sutured. The mice were maintained for an additional 2 weeks following the injection before being euthanized.

### Primary microglia isolation

The cerebral cortex was isolated from five P2 neonatal mice. The tissue was minced into ~1 mm³ pieces. Five milliliters of 0.25% trypsin was added, and the mixture was incubated at 37 °C for 15 min (the mixture was subsequently triturated ~10 times with a Pasteur pipette every 5 min during incubation). The digestion was terminated by adding complete culture medium, and the mixture was triturated ~30 times. The suspension was allowed to settle for 1 min to allow undigested tissue fragments to settle. The supernatant was filtered through a 40 µm cell strainer. Five milliliters of fresh medium was added to the sediment, triturated ~10 times, allowed to settle for 1 min, and the resulting supernatant was filtered through a cell strainer. The combined filtered cell suspension was centrifuged at 200×*g* for 10 min. The cell pellet was resuspended in complete medium and plated onto a poly-D-lysine (PDL)-coated T75 culture flask. This day is designated Day 0. After 2 days of culture (day 2), cell confluency and morphology were assessed. Astrocytes should be fully adherent, with microglia growing on top of the astrocyte layer. Either a half-medium change or a full medium change (after gentle rinsing with prewarmed PBS) was performed, depending on the amount of cellular debris present. The culture was continued for an additional 2–3 days. Monitor the number of microglia. If abundant microglia are observed, isolation is performed. If the number of microglia was insufficient, half of the medium was changed, and the culture was extended for an additional 2–3 days. Three milliliters of 0.05% trypsin was added to the T75 flask, which was subsequently incubated at 37 °C for 30 seconds. Trypsin was neutralized by adding an equal volume of complete medium. The microglia were detected by tapping the flask base. The supernatant was collected and centrifuged at 200×*g* for 10 min. The cell pellet was resuspended in complete medium and plated onto a 3 cm confocal dish. After the cells were allowed to stabilize in the incubator for 2 h, the nonadherent cells and debris were removed. The dish was gently rinsed with prewarmed PBS, and fresh complete medium was added. Once the microglia in the confocal dish are fully stabilized, the cells are fixed, and immunofluorescence staining with the microglial marker Iba1 is performed for identification. When the microglia in the confocal dish reached 70–90% confluence, they were transduced with AAV-IBA1-Fbxl12-EGFP at a multiplicity of infection (MOI) of 10⁴ genome copies (GCs) per cell. The medium was changed 24 h post transduction. At 72 h postinfection, the dish was stained with IBA1/FBXL12, and fluorescence was detected under a confocal microscope to confirm transduction efficiency before proceeding with subsequent cellular assays.

### Cell culture and treatment

Mouse SIM-A9 microglia and C8-D1A astrocytes were procured from the American Type Culture Collection (ATCC). SIM-A9 cells were cultured in Dulbecco’s modified Eagle’s medium (DMEM) supplemented with 10% fetal bovine serum and 5% donor horse serum. C8-D1A cells were cultured in Dulbecco’s modified Eagle’s medium (DMEM) supplemented with 10% fetal bovine serum. SIM-A9 ^Fbxl12−/−^, SIM-A9 ^Fbxl12 wild-type-overexpressing^ (Fbxl12^WT-OE^), and SIM-A9 ^Fbxl12 mutant-overexpressing^ (Fbxl12 ^Mut-OE^) strains were obtained from Cyagen Biosciences. The cells were seeded onto six-well plates at a density of 3 × 10^5^ cells/well and cultured in Dulbecco’s modified Eagle’s medium (DMEM) supplemented with 10% fetal bovine serum and 5% donor horse serum. Cells were treated with MBP and LPS, and C8-D1A cells were treated with MBP as a control. For prolonged MBP treatment, the cells were cultured in medium containing MBP and subcultured every 2 days to avoid massive proliferation. For cell viability analysis, a cell counting kit 8 (CCK8) solution was incubated with microglia for 2 h in the dark. The optical density (OD) was measured at 450 nm via an enzyme-labeling instrument.

### Transcriptome-seq of SIM-A9

RNA from SIM-A9 and Fbxl12^WT-OE^ cells was extracted and purified via a RNeasy Mini Kit (QIAGEN) according to the manufacturer’s instructions. The RNA quality and concentration were measured via a spectrophotometer (Thermo Fisher Scientific). Transcriptome sequencing was conducted at the Beijing Genomics Institute.

### Cell chemokine assay

Cell culture supernatant samples of SIM-A9 and Fbxl12^WT-OE^ cells were collected for testing. Two hundred microliters of blank cell culture medium sample was used as the experimental background control well (Blank) to eliminate sample background color interference.

### Lentiviral transduction for stable cell lines and RNA knockdown

Lentiviral vectors for FBXL12 (NM_013911.3) overexpression (LV-EFSKozak-FLAG tag/CMVEGFP/T2A/Puro) and FBXL12 T128A overexpression (LV-EFSKozak-FLAG tag/CMVmCherry/T2A/Puro) were generated via Cyagen. The lentiviral vectors were cotransfected with the packaging vectors psPAX2 and pMD2G into 293FT cells for lentivirus production. To generate stable cell lines, wild-type SIM-A9 and SIM-A9 ^Fbxl12−/−^ microglia were transduced with FBXL12-overexpressing and FBXL12 T128A-overexpressing lentiviruses with polybrene (Sigma) for 72 h, followed by treatment with 2 mg/mL puromycin for 4 days to select the cells. siRNA transfection was performed via Lipofectamine 3000 (Thermo Fisher Scientific). The siRNA oligonucleotide sequences used are listed in Supplementary Table 3.

### m6A-RNA immunoprecipitation (MeRIP) assay

The MeRIP assay was performed as previously described. Briefly, total RNA was extracted and treated with DNase (Sigma-Aldrich) to remove genomic DNA. After purification, the RNA was incubated with a m6A primary antibody. Dynabeads Protein G (Invitrogen) were washed and added to the mixture, followed by incubation for 2 h at 4 °C with rotation. m6A enrichment was determined via qRT‒PCR. The qPCR primers used are listed in Supplementary Table 2.

### Colony formation and cell proliferation assays

For colony formation, cells were seeded onto six-well plates at a density of 5 × 10^3^ cells/well, and three independent cultures under each condition were cultured for 72 h. The cells were washed with PBS, fixed with 4% PFA, and stained with crystal violet (Sigma‒Aldrich). Finally, the cell colonies were photographed and imaged via a microscope. For cell viability, the cells were seeded into 12-well plates at a density of 2 × 10^4^ cells/well. Cell viability was measured by counting the cells at different incubation times.

### Flow cytometry

Microglia were collected and fixed overnight in ice-cold 70% ethanol. Then, the microglia were stained with 50 mg/mL propidium iodide (Sigma-Aldrich) in the dark for 20 min at 4 °C and finally analyzed via flow cytometry (FACSCalibur, BD Biosciences) and software (Verity Software House).

### Transwell analysis

Microglia (5 × 10^4^) suspended in serum-free Dulbecco’s modified Eagle’s medium (DMEM) were added to the upper chamber of the inserts, which contained serum-free DMEM in the bottom chamber. After one hour of incubation at 37 °C, the medium in the bottom chamber was replaced with DMEM containing 10% fetal bovine serum and 5% donor horse serum for 14 h (the Fbxl12 knockout group was tested separately for 24 h), followed by gentle wiping with a cotton swab. Next, the cells in the lower chamber were fixed with 4% PFA for 10 min at room temperature and stained with crystal violet (Sigma) for 15 min. The cells were photographed and analyzed via a microscope.

### Microglial cell phagocytosis assay

Microglia were seeded on coverslips in 24-well plates at a density of 1 × 10⁵ cells per well. After 24 h, the culture medium was removed, and the cells were washed three times with phosphate-buffered saline (PBS). Following the manufacturer’s instructions, 1 mg/mL PHrodo™ Green *E. coli* BioParticles conjugate working solution was added to each well, and the cells were incubated at 37 °C for 10, 20, or 40 min. The cells were subsequently washed three times with PBS and fixed with 4% paraformaldehyde at room temperature for 15 min. After three additional PBS washes, the coverslips were mounted onto glass slides. Images were acquired via a Zeiss confocal microscope with a 488 nm excitation wavelength. For each sample, four random fields of view were captured. Phagocytic activity was quantified by measuring the fluorescence intensity of microglia that underwent phagocytosis (defined as cells whose mean fluorescence intensity exceeded background levels).

### Immunoprecipitation followed by LC‒MS/MS

Whole-cell lysates were incubated with 10 μg of rabbit anti-Fbxl12 antibody for 3 h at 4 °C. The IP samples were washed three times with lysis buffer and three times with PBS to eliminate detergent. Proteins eluted with 0.15% trifluoroacetic acid (TFA, Sigma) were adjusted to pH 8.0 and incubated with 5 mM DTT to reduce disulfide bonds. Protein samples were incubated with trypsin and CaCl_2_ overnight for digestion. The digested samples were incubated with formic acid and adjusted to pH 3.0, followed by centrifugation at 12,000×*g* for 5 min. The supernatant was gently loaded onto a C18 desalting column and washed thrice with 0.1% formic acid and 3% acetonitrile three times, followed by the addition of 0.1% formic acid and 70% acetonitrile for elution.

The separated peptides were analyzed by a Q ExactiveTM HF-X mass spectrometer (Thermo Fisher) with an ion source of Nanospray Flex™ (ESI, spray voltage of 2.1 kV, and ion transport capillary temperature of 320 °C). The 40 precursors with the highest abundance in the full scan were selected, fragmented by higher-energy collisional dissociation (HCD), and analyzed by MS/MS. Data analysis was conducted by Novogene Co. Ltd.

### AAV-FBXL12 production

An adeno-associated virus (AAV-PhP.eB) overexpression vector expressing FBXL12 under the control of the IBA1 promoter (AAV-Iba1>Kozak-FLAG-Mouse Fbxl12 CDS/P2A/EGFP) was generated by Cyagen. AAV plasmids carrying FBXL12 were cotransfected with pAAV-RC encoding the AAV genes rep and cap and the helper plasmids encoding E4, E24, and VA into AAV-293 cells for recombinant AAV generation. The AAV titer was 1 × 10^12^ GC/mL.

### Delivery of AAV-FBXL12 into adult mice

The mice were anesthetized 3 or 7 days after SCI via inhaled isoflurane (2%), which was delivered in oxygen-enriched air and subsequently secured on a stereotactic frame. The ninth thoracic vertebra was exposed using the center of the injury site pinned by a spinal clamp as the origin. A microliter syringe (Hamilton, 7633-01) was used to inject 1 × 10^12^ GC/ml AAV-FBXL12 in 1 μL of PBS at a rate of 200 nL/min at 1 mm rostral and caudal to the lesion (0.8 mm in depth), with one injection per site. Before moving to the next injection site, the needle was kept in place for 5 min to allow sufficient permeation of the viral mixture. The mice injected with empty plasmid AAV into the spinal cord adjacent to the injury site at 7 days postinjury composed the negative control group (NC group). After 2, 4, and 8 weeks, the animals were sacrificed, and the tissues were isolated.

For AAV-Mettl3 delivery, 7 days before SCI was induced, the T9–T10 vertebral plate of each mouse was opened, and AAV-METTL3 was injected following the same method as AAV-FBXL12 injection.

### Electrophysiological studies

Electrophysiological assays were performed eight weeks after the operation as described previously.^57^ MEPs can comprehensively reflect locomotor function in animals and indicate neurological recovery following SCI.

### Lesion analysis and quantification

The mice were euthanized and perfused 14, 28, or 56 days after SCI. The lesion site was defined and traced by staining for collagen I, collagen III, fibronectin, iNOS, S100A10, NF200, Sox2, GFAP, and 5-HT. The cell nuclei were stained with DAPI. The immunostaining intensity at the lesion site (1500 μm wide region at the epicenter) was measured via ZEISS software and normalized to that at the intact (proximal) region of the spinal cord.

### 10×Visium spatial transcriptomics tissue imaging, library preparation, and sequencing

Fresh tissues were washed with 1x PBS and subsequently embedded in OCT through a bath of dry ice. The RNA quality of the OCT-embedded blocks was assessed via an Agilent 2100 system. RNA integrity numbers of tissues greater than 7 were used for visual and spatial gene expression experiments. The cryosections were cut at a thickness of 10 μm and mounted onto the GEX arrays. The sections were placed on a Thermocycler Adaptor with the active surface facing up, incubated for 1 min at 37 °C, fixed for 30 min with methyl alcohol at −20 °C, and then stained with H&E (Eosin, Dako CS701; Hematoxylin Dako S3309; bluing buffer CS702). The brightfield images were taken on a Leica Aperio Versa8 whole-slide scanner at 20× resolution. The Visium Spatial Tissue Optimization Slide & Reagent Kit (10×Genomics, PN-1000193) was used to optimize the permeabilization conditions for the tissue according to the Visium Spatial Tissue Optimization User Guide (CG000238, 10×Genomics).

The tissue sections were subsequently subjected to gene expression analysis via a Visum spatial gene expression slide and Reagent Kit (10×Genomics, PN-1000184). cDNA amplification was performed on an S1000TM Touch Thermal Cycler (Bio-Rad). According to the manufacturer’s instructions, visual spatial libraries were constructed via a Visum spatial library construction kit (10× Genomics, PN-1000184). The libraries were finally sequenced via an Illumina NovaSeq 6000 sequencer with a sequencing depth of at least 50,000 reads per spot with a paired-end 150 bp reading strategy (performed by Beijing Novogene Technology Co., Ltd.).

### 10×Visium data analysis

The raw Visium spatial RNA-seq data and histological H&E images were processed via the Space Ranger pipeline (version 1.2.0, 10×Genomics) to align and summarize unique molecular identifier (UMI) counts according to the reference genome “Mouse Genome mm10”. The Seurat package (version 4.4.0) in R (version 4.4.1) was used for downstream visual analysis of the quantitative expression matrix output by Space Ranger. The expressed genes, UMI counts and mitochondrial levels were computed and filtered. A basic data quality assessment was performed using the following parameters: 200< count of identified features (nFeatures) < 5000 and mitochondrial gene expression percentages (percent.mito) <15%. Normalization was performed via SCTransform, and dimensional reduction was performed via principal component analysis. The Seurat objects of the SCI group and the AAV-FBXL12 delivery group were merged via Seurat CCA integration. Cell type definitions based on the marker list and pathway activity quantification were completed via the GSVA package (version 1.50.0) in R.

To allocate the cell type information in a comparable manner, we performed deconvolution analysis via conditional autoregressive-based deconvolution (CARD).^58^ The injured spinal cord scRNA-seq dataset was combined and used as a reference.^59^

### Statistical analysis

All the statistical analyses were performed via GraphPad Prism 8.0. The data are presented as the means ± standard errors of the means (SEMs) or ± square errors of the means (SDs). Unless otherwise stated, all experiments were performed in triplicate. Statistical significance was determined via one- or two-way analysis of variance (ANOVA).

## Supplementary information

Supplementary Materials

Video 1 WT microglia

Video 2 Fbxl12 overexpressing microglia

Video 3 mutant FBXL12 overexpression microglia

Video 4 FBXL12 ablation microglia

Video 5 Spinal cord rotating via X axis

Video 6 Spinal cord rotating via Y axis

Video 7 Motor function recovery of mice in AAV-FBXL12 delivery group

Video 8 BMS evaluation of SCI group mice

Video 9 BMS evaluation of AAV-Fbxl12 group mice

## Data Availability

The epitranscriptomic microarray data are available in the Gene Expression Omnibus (GEO) under accession GSE301251. The RNA-seq and spatial transcriptome sequencing data are deposited within the same project in the Genome Sequence Archive (GSA) under accession CRA027354. All supporting data are included in the manuscript and Supplementary Materials. Data will be shared upon reasonable request, subject to consent and data use restrictions.
