# Supplementary material for: F-box/LRR-repeat protein 12 reorchestrated microglia to inhibit scarring and achieve adult spinal cord injury repair
Source: Signal Transduct Target Ther. 2025 Aug 20;10:259. doi: 10.1038/s41392-025-02354-0 (PMC12365319; doi:10.1038/s41392-025-02354-0)
Supplement: Supplementary file 1 — Supplementary Materials [file 41392_2025_2354_MOESM1_ESM.docx]

**Supplementary Materials for**

F-box/LRR-repeat protein 12 reorchestrated microglia to inhibit scarring and achieve adult spinal cord injury repair

Xu Xu^1,2,#^, Feng Gao^1,2,#^, Qixin Chen^1,2,#^, Bairu Chen^1,2,#^, Wenyu Liang^1,2^, Runzhi Huang^1,2^, Yuchen Liu^1,2^, Zhibo Liu^1,2^, Yanjing Zhu^1,2^, Gufa Lin^1,2^, Bei Ma^1,2^, Letao Yang^1,2^, Shaorong Gao^2*^, Rongrong Zhu^1,2*^, Liming Cheng^1,2,3*^

# These authors contributed equally.

* Corresponding Authors:

LIMING CHENG: limingcheng@tongji.edu.cn;

RONGRONG ZHU: rrzhu@tongji.edu.cn;

SHAORONG GAO: gaoshaorong@tongji.edu.cn

**This File includes:**

Supplementary Figures 1-5

Supplementary Tables 1-3


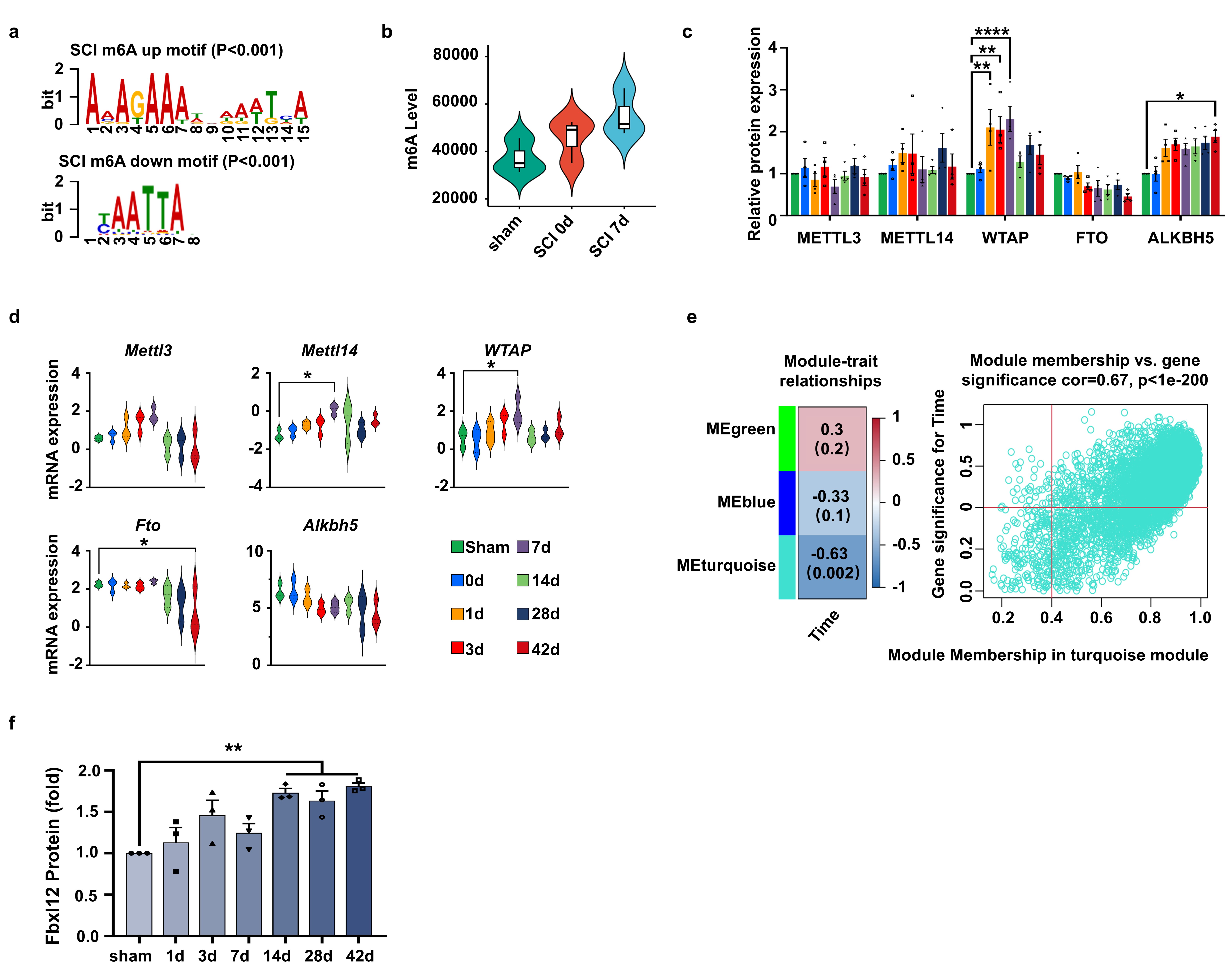


**Supplementary Figure 1. m6A methylation of Fbxl12 is a candidate master process during SCI progression. a.** Top up and down m6A motif base in spinal cord tissue after injury (t test, ****P<0.0001, n = 3). **b.** m6A level identified by epitranscriptomic microarray in spinal cord as indicated d.p.i (n = 3). **c.** Quantification of Immunoblotting as indicated in Figure 1c. Graph shows the blots indicate normalized β-actin (one-way ANOVA, mean ± SEM; * P<0.05, ** P<0.01, **** P<0.0001, n = 3). **d.** mRNA expression of writers and erasers of m6A modification identified by RNA-seq in spinal cord as indicated d.p.i (one-way ANOVA, * P<0.05, n = 3). **e.** Module-trait analysis from RNA-seq data. **f.** Quantification of Immunoblotting of FBXL12 in Figure 1j. Graph shows the blots indicate normalized β-actin (one-way ANOVA, mean ± SEM; ** P<0.01, n = 3).


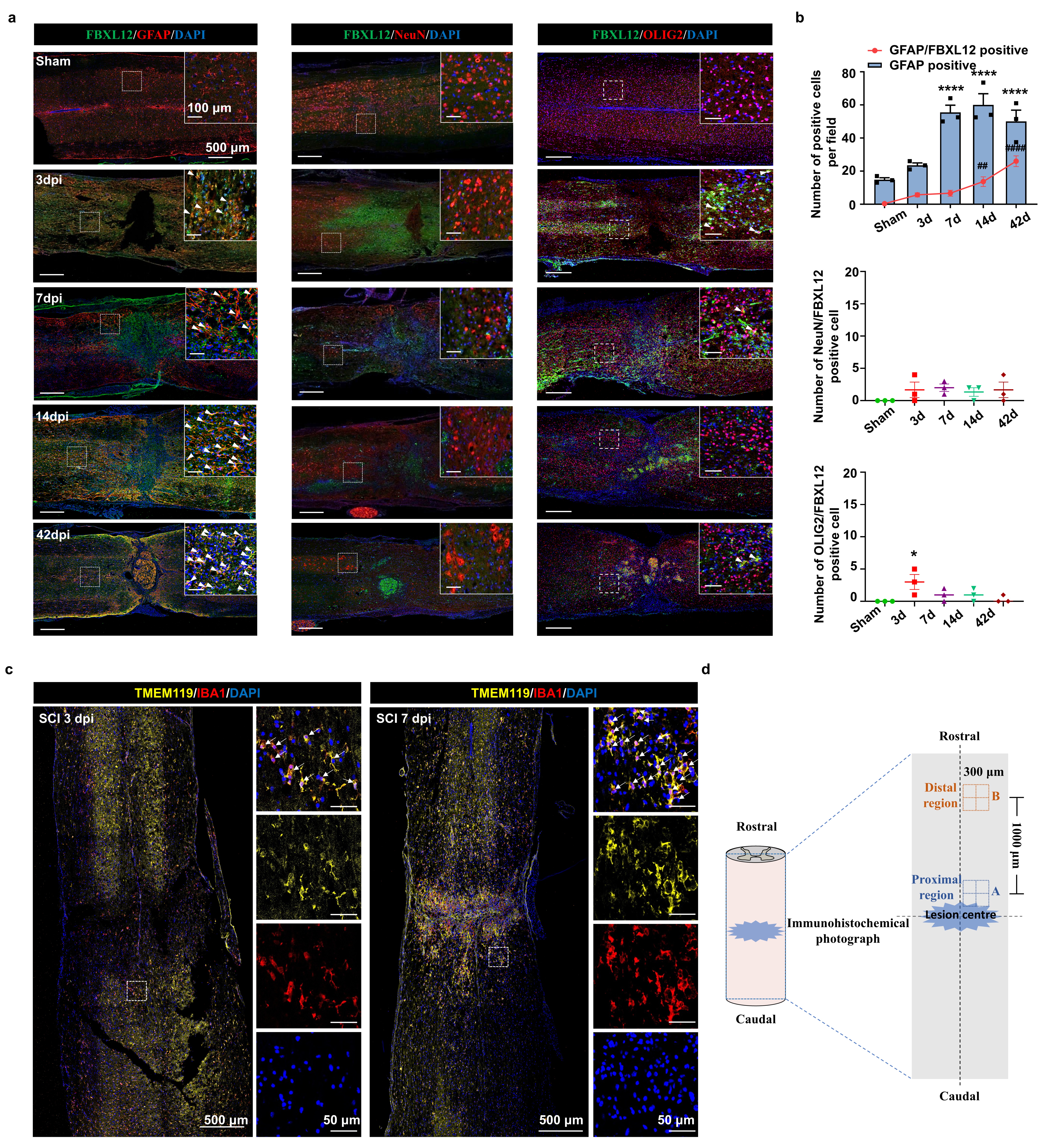


**Supplementary Figure 2. Expression analysis of Fbxl12 in different cell types following spinal cord injury.** **a.** Immunofluorescent stain of spinal cord tissue with FBXL12 and an astrocytes marker GFAP, neuron marker NeuN, and oligodendrocytes marker OLIG2. Rostral, caudal and scale bar as indicated. **b.** Quantification of GFAP and GFAP/FBXL12 positive cell within 500 µm range at different time points after injury (top), the quantification of NeuN/FBXL12 positive (middle) and OLIG2/FBXL12 positive (below) cells at different time points after spinal cord injury in **a**. (one-way ANOVA, mean ± SEM; * P<0.05, ** P<0.01, *** P<0.001, n = 3). **c.** Immunofluorescent stain of spinal cord tissue with TMEM119 and IBA1 at 3- and 7-day post injury. **d.** Schematic diagram of colocalization analysis of FBXL12 and IBA1 immunofluorescence in spinal cord tissue at 3-day post injury.

**
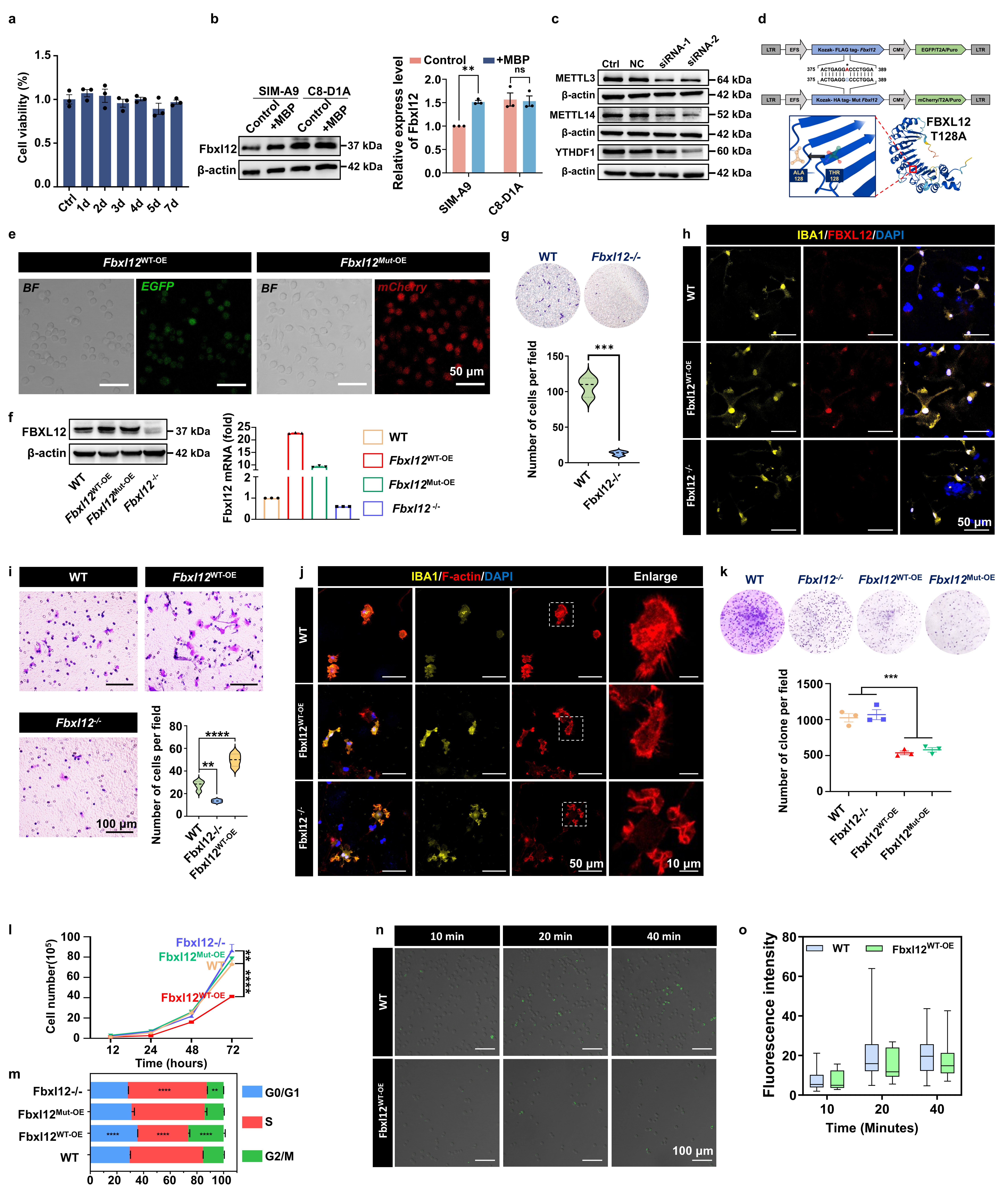
**

**Supplementary Figure 3. Fbxl12 regulates microglial proliferation, migration, cytoskeletal reorganization, and phagocytic functions. a.** Cell viability of SIM-A9 cells treated with MBP (200 ng/mL) for 7 days (n = 3). **b.** Immunoblotting of Fbxl12 in microglia and astrocytes with or without MBP treatment. Graph right shows the blots indicate normalized ß-actin (one-way ANOVA, mean ± SEM; ** P<0.01, n = 3). **c**. Immunoblotting of target protein in microglia. Where indicated, WT microglia were treated with siRNA of Mettl3, Mettl14 and YTHDF1. **d**. Schematic description of wildtype and mutant Fbxl12 constructs. **e.** Fluorescence photography of microglia with Fbxl12^WT-OE^ and Fbxl12^Mut-OE^ as indicated. **f.** Immunoblotting of Fbxl12 in different microglia. Graph blow shows the RT-qPCR quantitation of Fbxl12 mRNA expression as indicated cells (n = 3). **g.** Images of crystal violet stain of different microglia. Where indicated, cells were wildtype (WT), and Fbxl2 ablation (Fbxl12^-/-^). Graph right showed the quantification of migrated microglia. (t test, mean ± SEM; *** P<0.001, n = 3). **h**. Images of different primary microglia stained with FBXL12, IBA1 and DAPI. **i.** Images of crystal violet stain of different primary microglia. Graph below showed the quantification of migrated microglia. (one-way ANOVA, mean ± SEM; ** P<0.01, **** P<0.0001, n = 4). **j.** Images of different primary microglia stained with F-actin, IBA1 and DAPI. Representative primary microglia are shown in right magnified windows. Scale bar as indicated. **k.** Crystal violet stain of colony of microglia. Graph right shows quantification of colonies. (one-way ANOVA, mean ± SEM; *** P<0.001, n = 3). **l.** Cell number counting of microglia at indicated times. (two-way ANOVA, mean ± SEM; ** P<0.01, **** P<0.0001, n = 3). **m.** Cell cycle phases of different microglia. (two-way ANOVA, mean ± SEM; ** P<0.01, **** P<0.0001, n = 3, all groups compared with WT). **n.** Fluorescence photography of *E.coli* bioparticle as indicated time point. **o.** Quantification of bioparticles’ fluorescence in graph **n** (mean ± SEM, n = 30**)**.

**
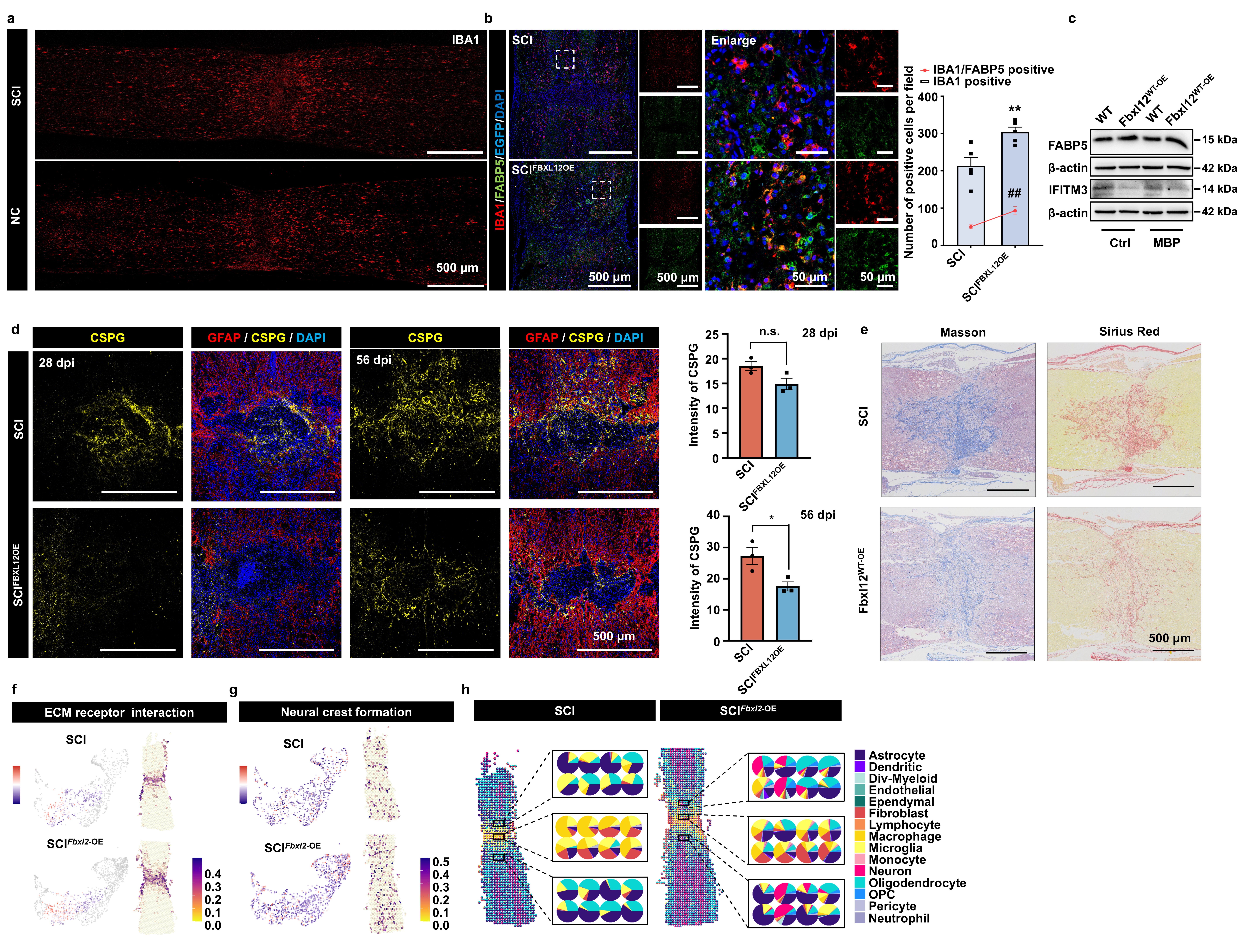
**

**Supplementary Figure 4. Target intrathecal delivery of FBXL12 regulates microglial states, reduces scarring and ameliorates pathology. a.** Images of spinal cord stained with IBA1 (4 mm in length centered around the injury site). **b.** Images of spinal sections at 28 dpi stained with antibodies against IBA1 and FABP5 (n = 5). Graph right showed the quantification of IBA1 and IBA1/FABP5 positive cell within 1500 µm range at different time points after injury. (t test, mean ± SEM; *, IBA1 positive, ** P<0.01, n = 5; #, IBA1/IFITM3 positive, ## P<0.01, n = 5). **c.** Immunoblotting of target protein in microglia. **d.** Images of spinal sections at 28 and 56 dpi stained with antibodies against the indicated proteins. Graph right showed quantification of the indicated immunoreactive area in the lesion site (t test, mean ± SEM; * P<0.05, n = 3, all groups compared with SCI). **e.** Images of Masson- and Sirius Rad stained spinal sections from different groups of mice at 28 dpi. **f.** UMAP plot in microglia with other cell types denoted by grey (left) and spatial distribution (right) of GSVA scores for ECM receptor interaction. **g.** UMAP plot (left) and spatial distribution (right) of GSVA scores for neural crest formation. **h.** The spatial view of the spots showing the proportions of different cell types as a pie chart in each spot.


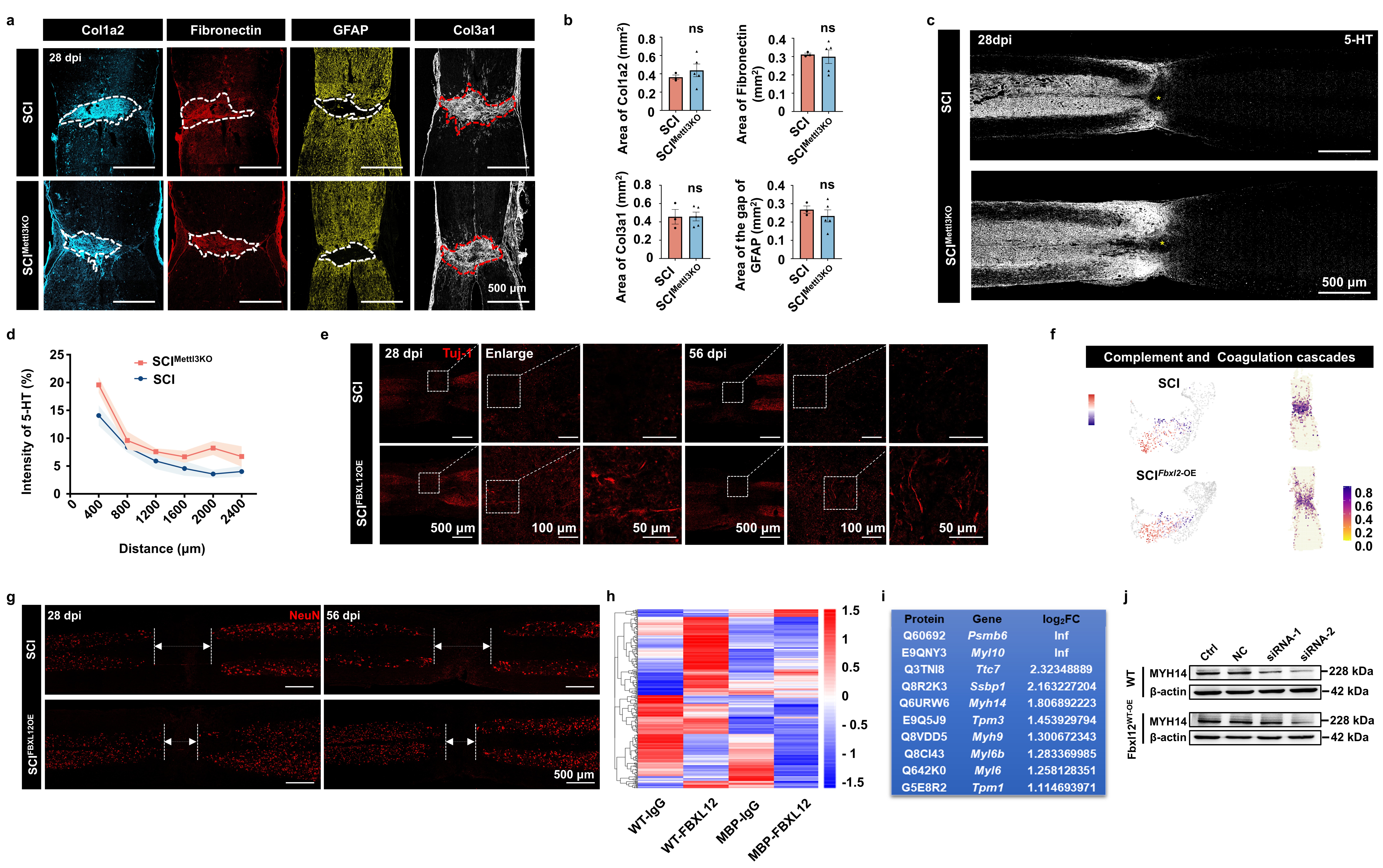


**Supplementary Figure 5. Intrathecal delivery of FBXL12 to microglia** **improves axon regeneration in mice. a.** Images of spinal sections at 28 dpi stained with antibodies against the indicated proteins. **b.** Quantification of the indicated immunoreactive area in the lesion site (dashed area in **a**) (t test, mean ± SEM; n = 3–5, all groups compared with SCI). **c.** Images of anti-5-HT-stained spinal sections from different groups of mice at 28 dpi, showing serotonergic axons. **d.** Quantification of the density of serotonergic axons (normalized to the density proximal to the lesion site) in spinal cord distal to the lesion site at 28 dpi (two-way ANOVA, mean ± SEM; n = 3–5). **e.** Images of spinal sections at 28 and 56 dpi stained with Tuj1. **f.** UMAP plot in microglia with other cell types denoted by grey (left) and spatial distribution (right) of GSVA scores for complement and coagulation cascades. **g.** Images of spinal cord at 28 and 56 dpi stained with NeuN (4 mm in length centered around the injury site). **h.** Heatmap of FBPs distribution from wildtype microglia and MBP treated microglia. **i.** The top 10 FBPs with significant changes in MBP treated microglia, compared with wildtype microglia. **j.** Immunoblotting of target protein in microglia. Where indicated WT and Fbxl12^WT-OE^ microglia treated with siRNA of MYH14 for 24 h.

**Supplementary Table 1: Reagent or Resource**

| **REAGENT or RESOURCE** | **SOURCE** | **IDENTIFIER** |
| --- | --- | --- |
| **Antibodies** | | |
| Rabbit anti-FbxL12 | Abcam | Cat#ab96831 |
| Rabbit anti-FbxL12 | Invitrogen | Cat#PA5-64887 |
| Rabbit anti-FbxL12 | ABclonal | Cat#A14589 |
| Goat anti-FbxL12 | Novus | Cat#NB100-1295 |
| Rabbit anti-m6A | Synaptic System | Cat#202 003 |
| Rabbit anti-METTL3 | Abcam | Cat#ab195352 |
| Mouse anti-METTL14 | Abcam | Cat#ab220030 |
| Rabbit anti-WTAP | Abcam | Cat#ab155655 |
| Mouse anti-ALKBH5 | Proteintech | Cat#67811-1-lg |
| Mouse anti-FTO | Proteintech | Cat#68111-1-lg |
| Rabbit anti-IBA1 | FUJIFILM Wako | Cat#019-19741 |
| Guinea pig anti-IBA1 | Synaptic System | Cat#HS-234 308 |
| Chicken anti-GFAP | Abcam | Cat#ab4674 |
| Mouse anti-NeuN | Abcam | Cat#ab104224 |
| Guinea pig anti-NeuN | Sigma-Aldrich | Cat#ABN90 |
| Rabbit anti-Olig2 | Abcam | Cat#ab109186 |
| Mouse anti-YTHDF1 | Proteintech | Cat#66745-1-lg |
| Rabbit anti-YTHDF3 | Proteintech | Cat#25537-1-AP |
| Mouse anti-MYH9 | Proteintech | Cat#60233-1-lg |
| Rabbit anti-MYH14 | Proteintech | Cat#20716-1-AP |
| Mouse anti-Beta Actin | Proteintech | Cat#66009-1-lg |
| Rabbit anti-K63-linkage Specific Polyubiquitin | ABclonal | Cat#A18164 |
| Rabbit anti-FABP5 | Proteintech | Cat#12348-1-AP |
| Rabbit anti-IFITM3 | Proteintech | Cat#11714-1-AP |
| Mouse anti-Col1a2 | Santa Cruz | Cat#sc-393573 |
| Rabbit anti-Col3a1 | Proteintech | Cat#22734-1-AP |
| Rabbit anti-iNOS | Proteintech | Cat#18985-1-AP |
| Goat anti-S100A10 | R&D | Cat#AF2655377 |
| Rabbit anti-Fibronectin | Millipore | Cat#AB2033 |
| Rabbit anti-NF200 | Sigma-Aldrich | Cat#N4142 |
| Chicken anti-Nestin | Abcam | Cat#ab134017 |
| Rabbit anti-SOX2 | Abcam | Cat#ab97959 |
| Goat anti-5-HT | IMMUNOSTAR | Cat#20079 |
| Rabbit anti-5-HT | IMMUNOSTAR | Cat#20080 |
| Mouse anti-CSPG | Sigma-Aldrich | Cat#C8035 |
| Mouse anti- CD206 | BioLegend | Cat#141707 |
| Rabbit anti-TMEM119 | ABclonal | Cat#A27143 |
| Mouse anti-Tubulin β 3 | BioLegend | Cat#801209 |
| Alexa Fluor 647-conjugated Streptavidin | Invitrogen | Cat#S32357 |
| Alexa Fluor 488-conjugated donkey anti rabbit/goat | Invitrogen | Cat#A32790/A32814 |
| Alexa Fluor 594-conjugated anti rabbit | Cell Signaling Techonology | Cat#8889S |
| Alexa Fluor 647-conjugated donkey anti mouse/rabbit/goat | Invitrogen | Cat#A32787/A32795/A32849 |
| Alexa Fluor 647 AffiniPure Donkey Anti-Guinea Pig IgG (H+L) | Jackson ImmunoResearch | Cat#706-605-148 |
| Alexa Fluor 594 AffiniPure Donkey Anti-Chicken IgG (H+L) | Jackson ImmunoResearch | Cat#703-585-155 |
| Alexa Fluor 647 AffiniPure Donkey Anti-Chicken IgG (H+L) | Jackson ImmunoResearch | Cat#703-605-155 |

**Chemicals, Peptides, and Recombinant Proteins**

| MBP | Sigma | Cat#M2295 |
| --- | --- | --- |
| LPS | Sigma | Cat#L3012 |
| Lipofectamine™ 3000 | Invitrogen | Cat#L3000015 |
| Crystal violet | Sigma | Cat#C0775 |
| pHrodo™ Green E. coli BioParticles™ | Invitrogen | Cat#P35366 |

**Critical Commercial Assays**

| m6A MeRIP Kit | GenSeq | Cat#GS-ET-001 |
| --- | --- | --- |
| Actin-Tracker Red-555 | Beyotime | Cat#C2203S |
| Masson Trichrome Staining Kit | Solarbio | Cat#G1340 |
| Picro Sirius Red Stain Kit | Solarbio | Cat#S8060-5 |

**Experimental Models: Cell Lines**

| SIM-A9 | ATCC | CRL-3216 |
| --- | --- | --- |
| C8-D1A | ATCC | CRL-2541 |

**Supplementary Table 2: Primers used for qPCR.**

| Mouse-Fbxl12 | forward: CTGCCTCACTATGCCCAAGC  reverse: GCAAGCCCTGACGATGACC |
| --- | --- |
| Mouse-GAPDH | forward: AAGAAGGTGGTGAAGCAGG  reverse: GAAGGTGGAAGAGTGGGAGT |

**Supplementary Table 3: RNAi oligonucleotides sequences.**

|  | sense (5’-3’) | antisense (5’-3’) |
| --- | --- | --- |
| Human-Fbxl12 | CGAUGCGACCUAAAGUCAUTT | AUGACUUUAGGUCGCAUCGTT |
| Mouse-Mettl3-1 | CCUCAGUGGAUCUGUUGUGAUTT | AUCACAACAGAUCCACUGAGGTT |
| Mouse-Mettl3-2 | GCACCCGCAAGAUUGAGUUAUTT | AUAACUCAAUCUUGCGGGUGCTT |
| Mouse-Mettl14-1 | CGGGAAAGAAACCGAUCCAAUTT | AUUGGAUCGGUUUCUUUCCCGTT |
| Mouse-Mettl14-2 | CUAUGAUACAUCUGCUCCAAATT | UUUGGAGCAGAUGUAUCAUAGTT |
| Mouse-MYH14-1 | CACCGACAUCAUAGUGUCUUUTT | AAAGACACUAUGAUGUCGGUGTT |
| Mouse-MYH14-2 | GUUUGGCAACAUUGUCCUGAATT | UUCAGGACAAUGUUGCCAAACTT |
| Mouse-YTHDF1-1 | GCUGAAGAUUAUCGCUUCCUATT | UAGGAAGCGAUAAUCUUCAGCTT |
| Mouse-YTHDF1-2 | GCCCACAGCUAUAACCCUAAATT | UUUAGGGUUAUAGCUGUGGGCTT |
| Mouse-YTHDF3-1 | CCAAUAACCAAUUACGACAUATT | UAUGUCGUAAUUGGUUAUUGGTT |
| Mouse-YTHDF3-2 | GCCAGACAAAUCAGAAAUAUATT | UAUAUUUCUGAUUUGUCUGGCTT |
